# The two alternative NADH:quinone oxidoreductases from *Staphylococcus aureus*: two players with different molecular and cellular roles

**DOI:** 10.1128/spectrum.04152-23

**Published:** 2024-07-16

**Authors:** Filipa V. Sena, Filipe M. Sousa, Ana R. Pereira, Teresa Catarino, Eurico J. Cabrita, Mariana G. Pinho, Francisco R. Pinto, Manuela M. Pereira

**Affiliations:** 1 Department of Chemistry and Biochemistry, Faculty of Sciences, University of Lisbon, Lisboa, Portugal; 2 BioISI-Biosystems & Integrative Sciences Institute, Faculty of Sciences, University of Lisbon, Lisboa, Portugal; 3 Instituto de Tecnologia Química e Biológica António Xavier, Universidade Nova de Lisboa, Oeiras, Portugal; 4 Departamento de Química, Faculdade de Ciências e Tecnologia, Universidade Nova de Lisboa, Caparica, Portugal; 5 UCIBIO, Departamento de Química, Faculdade de Ciências e Tecnologia, Universidade Nova de Lisboa, Caparica, Portugal; Center of Innovative and Applied Bioprocessing, Mohali, Punjab, India

**Keywords:** respiratory chain, NAD(P)H, quinones, charge-transfer complex, membrane proteins, monotopic proteins, alternative NADH oxidase

## Abstract

*Staphylococcus aureus* is an opportunistic pathogen that has emerged as a major public health threat due to the increased incidence of its drug resistance. *S. aureus* presents a remarkable capacity to adapt to different niches due to the plasticity of its energy metabolism. In this work, we investigated the energy metabolism of *S. aureus*, focusing on the alternative NADH:quinone oxidoreductases, NDH-2s. *S. aureus* presents two genes encoding NDH-2s (NDH-2A and NDH-2B) and lacks genes coding for Complex I, the canonical respiratory NADH:quinone oxidoreductase. This observation makes the action of NDH-2s crucial for the regeneration of NAD^+^ and, consequently, for the progression of metabolism. Our study involved the comprehensive biochemical characterization of NDH-2B and the exploration of the cellular roles of NDH-2A and NDH-2B, utilizing knockout mutants (Δ*ndh-2a* and Δ*ndh-2b*). We show that NDH-2B uses NADPH instead of NADH, does not establish a charge-transfer complex in the presence of NADPH, and its reduction by this substrate is the catalytic rate-limiting step. In the case of NDH-2B, the reduction of the flavin is inherently slow, and we suggest the establishment of a charge transfer complex between NADP^+^ and FADH_2_, as previously observed for NDH-2A, to slow down quinone reduction and, consequently, prevent the overproduction of reactive oxygen species, which is potentially unnecessary. Furthermore, we observed that the lack of NDH-2A or NDH-2B impacts cell growth, volume, and division differently. The absence of these enzymes results in distinct metabolic phenotypes, emphasizing the unique cellular roles of each NDH-2 in energy metabolism.

**IMPORTANCE:**

*Staphylococcus aureus* is an opportunistic pathogen, posing a global challenge in clinical medicine due to the increased incidence of its drug resistance. For this reason, it is essential to explore and understand the mechanisms behind its resistance, as well as the fundamental biological features such as energy metabolism and the respective players that allow *S. aureus* to live and survive. Despite its prominence as a pathogen, the energy metabolism of *S. aureus* remains underexplored, with its respiratory enzymes often escaping thorough investigation. *S. aureus* bioenergetic plasticity is illustrated by its ability to use different respiratory enzymes, two of which are investigated in the present study. Understanding the metabolic adaptation strategies of *S. aureus* to bioenergetic challenges may pave the way for the design of therapeutic approaches that interfere with the ability of the pathogen to successfully adapt when it invades different niches within its host.

## INTRODUCTION

*Staphylococcus aureus* is a natural inhabitant of the skin and mucous membrane of humans. It is an opportunistic pathogen and one of the most frequent causes of community-acquired and nosocomial infections. This Gram-positive bacterium has become a major public health threat due to the increased incidence of its drug resistance. Despite its prominence as a pathogen, the energy metabolism of *S. aureus* remains underexplored, with its respiratory enzymes often escaping thorough investigation. *S. aureus* bioenergetic plasticity is illustrated by its ability to use several carbon sources in either aerobic or anaerobic respiration, with oxygen or nitrate as the respective electron acceptors. In addition, it may also ferment in the absence of external electron acceptors, providing the presence of a rich carbon source, such as glucose (1–3). *S. aureus* contains genes encoding various quinone reductases, playing a crucial role in connecting multiple catabolic pathways and the respiratory chain. This ensures versatility and robustness to the bacterial energy metabolism (4–9), which allows *S. aureus* to survive and proliferate in multiple host niches.

The most crucial quinone reductases are NADH:quinone oxidoreductases because they connect almost all catabolic pathways to respiratory chains. Three enzymes may perform this reaction: respiratory Complex I, type 2 NADH:quinone oxidoreductase (NDH-2), and Na^+^-translocating NADH:quinone oxidoreductase (Nqr). Because *S. aureus’* genome only contains genes encoding NDH-2s, these enzymes play a pivotal role in the regeneration of NAD^+^ and the subsequent functioning of the catabolic pathways (7–9). NDH-2s are monotopic proteins that, although performing the same catalytic reaction as respiratory Complex I, do not translocate charges across the membrane (10, 11) and thus do not contribute directly to the establishment of the membrane potential. The study of NDH-2s from several organisms, including from *S. aureus*, has already been addressed (7–10, 12–21). *S. aureus*, as most of the organisms belonging to the Bacillales order, presents two genes encoding two NDH-2s, NDH-2A (SAOUHSC_00878) and NDH-2B (SAOUHSC_00875) (4, 5). We previously performed a thorough functional and structural characterization of NDH-2A, which included obtaining its crystal and solution structures (9, 20–22). We showed NDH-2A has different binding sites for the two substrates and establishes a charge-transfer (CT) complex between NAD^+^ and the flavin, which is dissociated by the quinone. These findings indicated the involvement of a ternary complex in the catalytic mechanism (9) and resolved the controversy whether the substrates bind to the same site in NDH-2 or in different places relative to the flavin (13–15). Furthermore, we proposed that the CT complex is crucial for the overall mechanism of NDH-2A and may play a role in maintaining a low quinol/quinone ratio, thereby avoiding excessive reactive oxygen species production *in vivo* (21). Schurig-Briccio and co-workers (7, 8) suggested that the two enzymes, NDH-2A (ndhC, SAB0807 *S. aureus* RF122 strain) and NDH-2B (ndhF, SAB0804c *S. aureus* RF122 strain), exhibit the same catalytic activity, NADH:quinone oxidoreductase. However, they observed distinct phenotypes upon the knockout of their respective coding genes.

In this study, we investigated NDH-2B and demonstrated that it functions preferentially as a NADPH:quinone oxidoreductase and at pH 5.5, indicating a catalytic function distinct from that of NDH-2A. Additionally, we explored the cellular roles of the two NDH-2s by assessing the influence of their absence on cell growth, the cell cycle, and the metabolism of *S. aureus*. We observed that the absences of Ndh-2A and Ndh-2B impact differently on energy metabolism, further supporting that each enzyme plays unique cellular roles in *S. aureus* (Fig. S1).

## RESULTS

### Biochemical characterization of NDH-2B from *Staphylococcus aureus*

The *ndh-2b* gene was successfully expressed in *Escherichia coli*, and the resultant protein was purified from the membrane fraction using a two-step chromatographic process involving Q-sepharose and Superdex 200 columns. Only one protein band corresponding to the predicted molecular mass of NDH-2, approximately 40 kDa, was discernible in SDS-PAGE (Fig. S2). Through blue native-PAGE analysis, NDH-2B was established as a monomer in solution, indicated by the presence of only a single band below the 66 kDa marker (Fig. S2). The UV-visible absorption spectrum of the purified NDH-2B exhibited a band with maximum absorption at 450 nm, consistent with the presence of a flavin in its oxidized state. Subsequent HPLC analysis confirmed the flavin as flavin adenine dinucleotide (FAD), and its reduction potential was determined to be −202 ± 20 mV vs SHE, using cyclic voltammetry. NDH-2B demonstrated stability up to 40°C, in different buffers, as evidenced by the change in FAD fluorescence in thermal denaturation assays.

**Fig 1:**
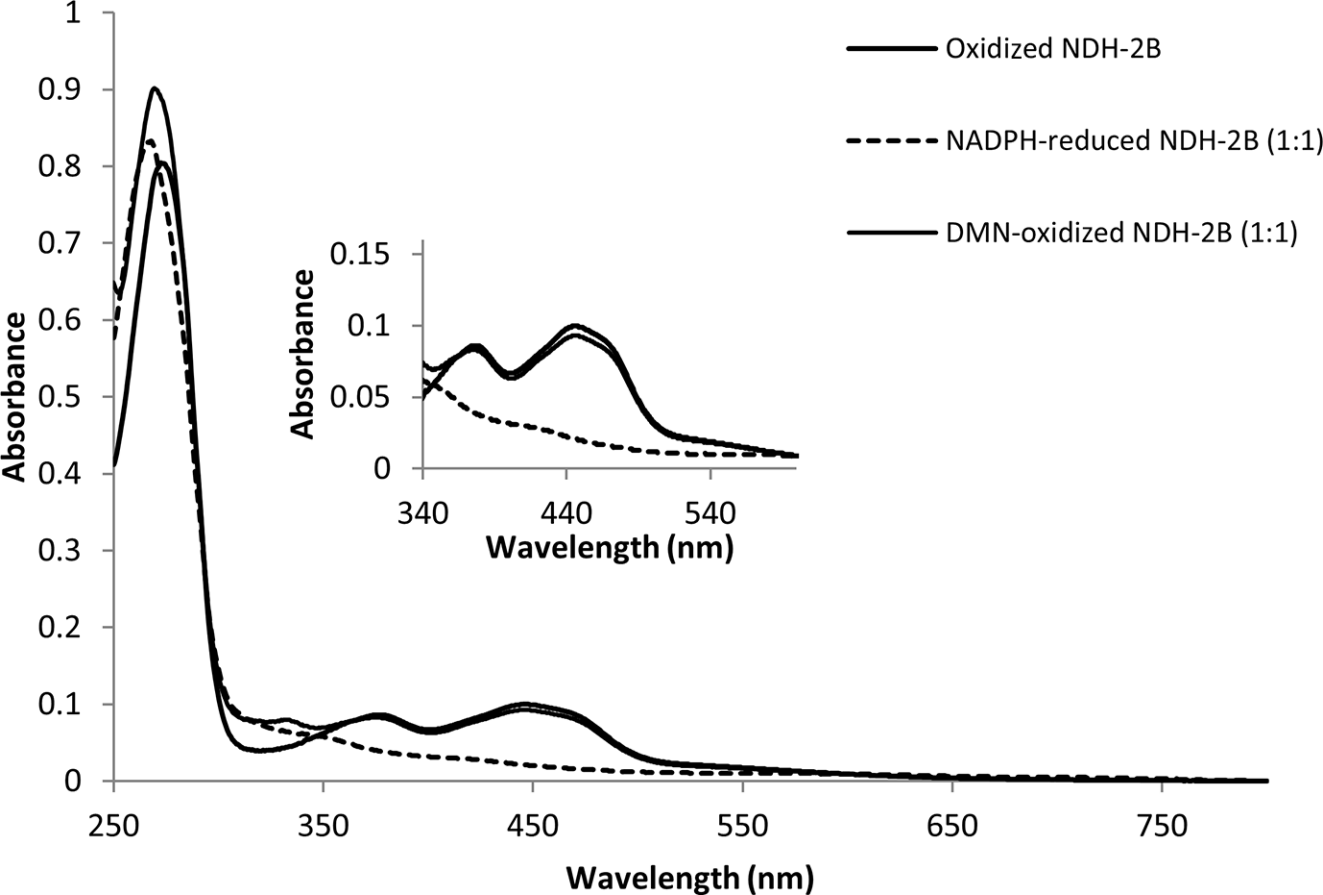
UV-visible spectra of NDH-2B from *S. aureus*. Purified NDH-2B in oxidized state (solid line), reduced with NADPH in a 1:1 ratio (dashed line), and oxidized by DMN (1:1) after NADPH reduction (dotted line). The inset figure expands the absorption spectra in the 340–550 region.

NDH-2B was isolated in its oxidized state and its reduction state was achieved under anaerobic conditions by the addition of NADPH in 1:1 stoichiometry (Fig. 1). The reduced state is characterized by the loss of absorbance in the 325–500 nm region of the UV-visible spectrum. Remarkably, the spectrum of the reduced protein did not exhibit any absorbance band in the visible region, particularly around 650–800 nm as observed for the NADH-reduced NDH-2A (9, 21). Such a band is typical of the establishment of a CT complex between NAD^+^ and the reduced flavin (9). Thus, in the case of NDH-2B, there is no evidence for the presence of such a complex. Further incubation with a stoichiometric amount of the quinone substrate, 2,3-dimethyl-1,4-naphthoquinone (DMN), leads to reoxidation of the enzyme, as observed by the reappearance of the typical absorbance band of the flavin (Fig. 1).

### NDH-2B is a NADPH:quinone oxidoreductase

**Fig 2:**
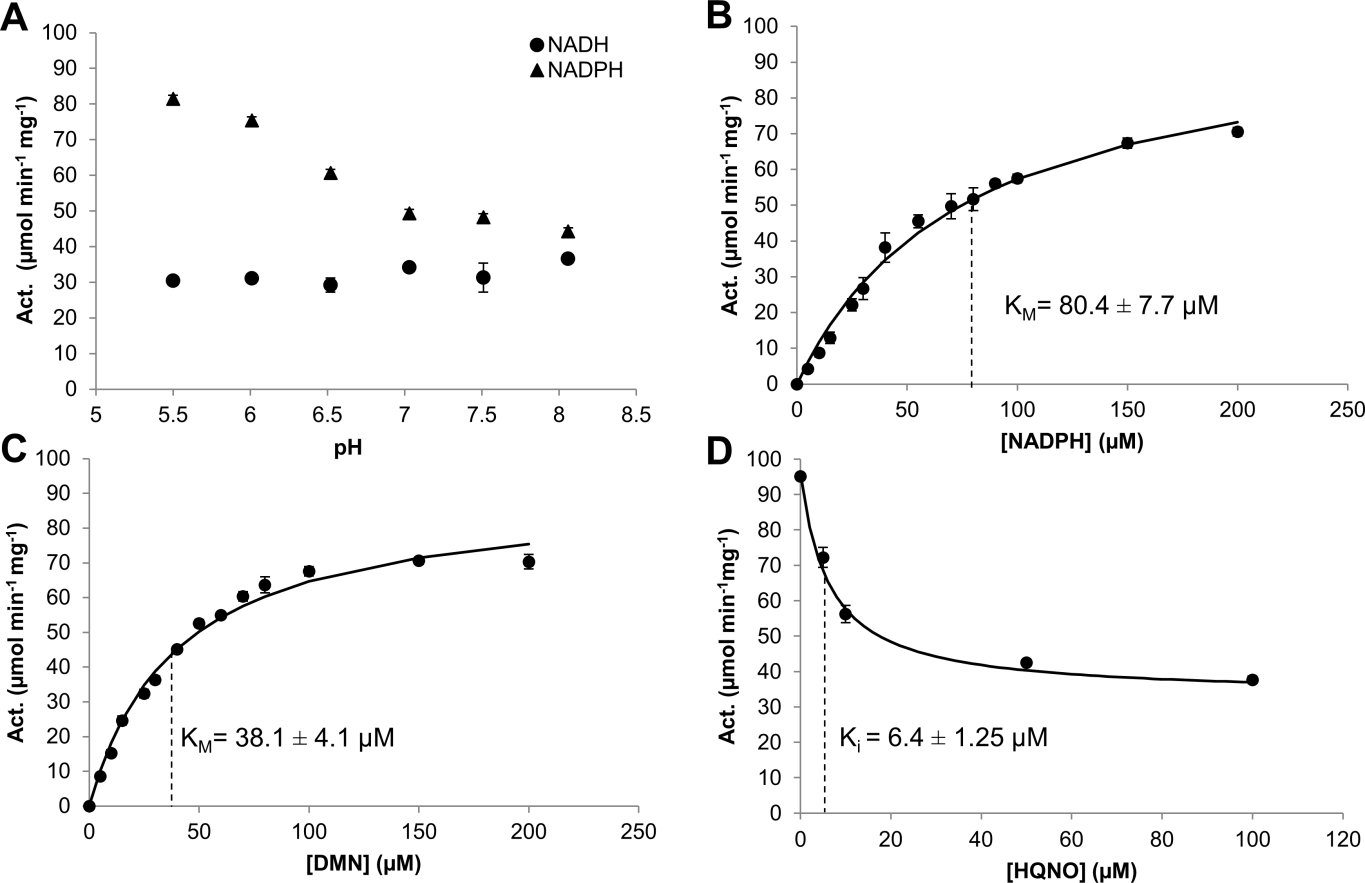
Enzyme activity of NDH-2B from *S. aureus*. (**A**) pH dependence of *S. aureus* NDH-2B enzyme activity. NAD(P)H:DMN oxidoreduction [NADH (dots) and NADPH (triangles)] measured in an anaerobic chamber at 35°C with 150 µM DMN, 200 µM NADH or NADPH, 0.2 µM NDH-2B, and different pH buffer solutions of 50 mM MES Bis-Tris-Propane, 250 mM NaCl. (**B**) NADPH:quinone oxidoreductase activity varying the concentration of NADPH. (**C**) NADPH:quinone oxidoreductase activity varying the concentration of DMN. (**D**) Steady-state analysis of *S. aureus* NDH-2B activity in the presence of 2-n-heptyl-4-hydroxyquinoline-N-oxide (HQNO). In panels **B–D**, the protein concentration used was 0.2 µM in 50 mM MES Bis-Tris-Propane, 250 mM NaCl buffer pH 5.5. The black dots representing the experimental data were fitted with the Michaelis-Menten equation (**B**) or (**C**) with equation $v_{0}^{i}=v_{0}^{max}\frac{\left(1+\beta \frac{\left[I\right]}{K_{i}^{app}}\right)}{\left(1+\frac{\left[I\right]}{K_{i}^{app}}\right)}$ (**D**). In all cases presented, the values are the median of at least three independent experiments, and bars represent the standard mean deviation.

Maximal activity for NDH-2B occurred at pH 5.5 with NADPH as a substrate, with a specific activity of 81.45 ± 1.05 µmol min^−1^ mg^−1^ (Fig. 2A). The activity of the enzyme using NADH as a substrate was almost constant (~30 µmol min^−1^ mg^−1^) for all pH values tested and always lower than when using NADPH as a substrate. This contrasts with what we observed for NDH-2A, which has nearly no activity when NADPH is the substrate instead of NADH and presents the highest activity at pH 7 (Table 1).

**TABLE 1:** Comparison of kinetic parameters for NDH-2A and NDH-2B

Steady state kinetics	NDH-2A	NDH-2B
Specific activity (µmol NADH oxidized min^−1^ mg^−1^), pH 7.0	131.1 ± 2.7^*a*^	34.03 ± 0.86
Specific activity (µmol NADH oxidized min^−1^ mg^−1^), pH 5.5	122.10 ± 18.30	30.37 ± 1.05
Specific activity (µmol NADPH oxidized min^−1^ mg^−1^), pH 7.0	2.81 ± 1.44	49.24 ± 3.56
Specific activity (µmol NADPH oxidized min^−1^ mg^−1^), pH 5.5	19.65 ± 2.06	81.45 ± 1.05
*k*_cat_ s^−1^	128.96 (pH 7.0)^*a*^	67.51 (pH 5.5)
*K*_*M*_ NAD(P)H (µM)	20.5 ± 0.3 (pH 7.0)^*a*^	80.4 ± 7.7 (pH 5.5)
*K*_*M*_ DMN (µM)	69.9 ± 6.4 (pH 7.0)^*a*^	38.1 ± 4.1 (pH 5.5)

^*a*^ Previously determined (9).

We determined the kinetic parameters for NDH-2B in the presence of DMN by varying the concentration of NADPH from 5 to 200 µM. The enzyme activity (*V*_max_) obtained was 102.8 ± 2.5 µmol min^−1^ mg^−1^, and the Michaelis-Menten constant (*K*_*M*_) obtained was 80.4 ± 7.7 µM (Table 1; Fig. 2B). The kinetic parameters for NDH-2B in the presence of NADPH by varying the concentration of DMN from 0 to 200 µM were 38.1 ± 4.1 µM, *K*_*M*_, and 88.0 ± 3.8 µmol min^−1^ mg^−1^, *V*_max_ (Table 1; Fig. 2C). The enzyme presented a slightly lower *K*_*M*_ value for DMN than for NADPH, indicating that it has a higher affinity for DMN compared to NADPH. A *K*_*i*_^app^ value of 6.4 ± 1.25 µM for 2-n-heptyl-4-hydroxyquinoline-N-oxide (HQNO), a quinone mimicking inhibitor, was obtained for NADPH:DMN oxidoreductase activity (Fig. 2D).

### NDH-2B does not establish a charge-transfer complex and the rate-limiting step is NADPH oxidation

Pre-steady-state kinetics assays with NDH-2B were performed inside an anaerobic chamber in the presence of a scavenging system to ensure oxygen levels were as low as possible. The temperature was maintained at 18°C. For the study of the reductive half reaction, 10 µM NDH-2B was mixed with 20 µM NADPH (1:2 ratio) at pH 5.5. The oxidized fraction was calculated from the spectra and plotted against time. The reduction rate constant 0.86 ± 0.02 s^−1^ was obtained from the fit of the experimental data with an exponential curve (Fig. S3). For the study of the oxidative half reaction, 10 mM NADPH-reduced NDH-2B was mixed with 30 µM DMN (1:3 ratio) at pH 5.5. The oxidation rate constant 6.7 ± 0.3 s^−1^ was obtained from the fit of the kinetic traces acquired at 450 nm with an exponential curve (Fig. S3). A multiple turnover experiment was also performed. Under these conditions, the reduction of NDH-2B could only be observed when NADPH was in excess and after all DMN was consumed. The reduction rate constant obtained with 10 µM NDH-2B in the presence of 50 µM NADPH and 30 µM DMN (1:5:3 ratio) was 0.72 ± 0.01 s^−1^. Since the rate constant of the oxidative half reaction is one order of magnitude higher than the rate constant of the reductive half reaction, we may conclude that the rate-limiting step is NADPH oxidation. This result contrasts with that observed for NDH-2A, for which the limiting step is quinone reduction. However, for NDH-2A, we observed the establishment of a CT complex that slowed down the reduction of the quinone (9, 21). In the absence of a CT complex, reduction of the quinone could occur faster.

### The two substrates bind at distinct sites in NDH-2B

**Fig 3:**
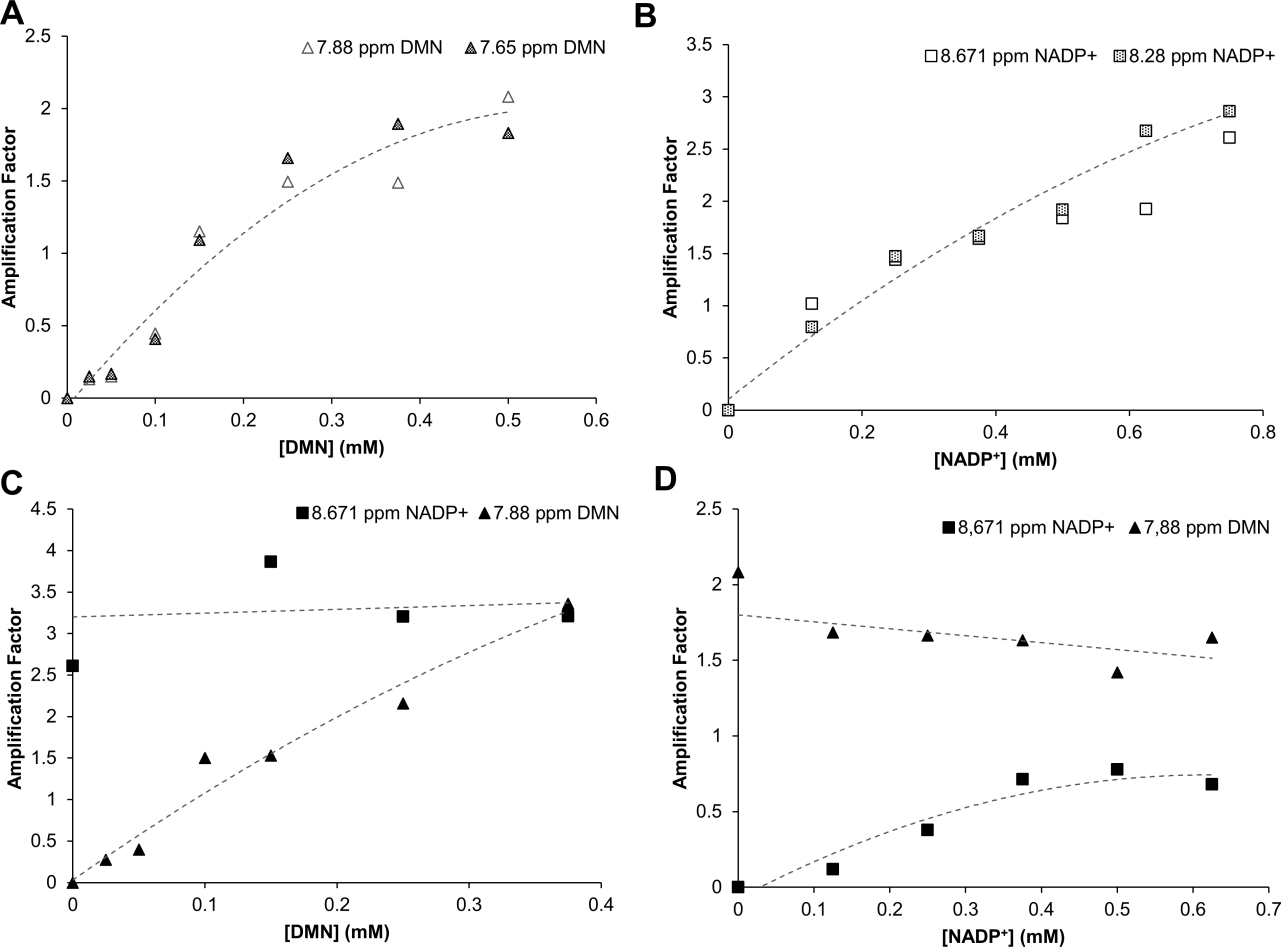
NDH-2B-substrate interaction monitored by STD-NMR. (**A**) Plot of the STD amplification factor of DMN (from signals at 7.88 and 7.65 ppm) vs [DMN] measured in NDH-2B solutions titrated with DMN. (**B**) Plot of the STD amplification factor of NADP^+^ (from signals at 8.671 and 8.28 ppm) vs [NADP^+^] measured in NDH-2B solutions titrated with NADP^+^. (**C**) Plot of the STD amplification factor of DMN (from signals at 7.88 and 7.65 ppm) vs [DMN] measured in NDH-2B solutions titrated with DMN in the presence of NADP+ for which the NADP^+^ amplification factor in the same sample was also measured. (**D**) Plot of the STD amplification factor of NADP^+^ (from signals at 8.671 and 8.28 ppm) vs [NADP^+^] measured in NDH-2B solutions titrated with NADP^+^ in the presence of DMN for which the DMN amplification factor in the same sample was also measured. The protein concentration used was 10 µM. The dotted curves are meant to be visual guidelines.

The interactions of DMN and NADP^+^ with NDH-2B were studied by saturation transfer difference (STD) NMR spectroscopy, measuring the level of saturation transfer from the protein to the ligands when the protein is selectively saturated by magnetization. NADP^+^ was chosen instead of NADPH to avoid the occurrence of the catalytic reaction and the consequent changes in NADPH and NADP^+^ signals due to substrate consumption. Two STD-NMR signals (7.88 and 7.65 ppm) were detected corresponding to the hydrogen atoms of the aromatic rings of the quinone (Fig. S4A). These two signals showed relative saturation percentages of 92% and 96%, revealing that contact with the protein is probably occurring through these groups. With the increase in DMN concentration, the integral of the signals corresponding to the DMN peaks also increased (Fig. 3A). In the case of NADP^+^, five signals were detected (9.2, 9.0, 8.7, 8.2, and 6.0 ppm), which correspond to the aromatic ring of the nicotinamide (signals 1, 2, and 3, see Fig. S4B), and have relative saturation percentages in the range of 27%–46%. The signals at 8.2 and 6.0 ppm correspond to the adenine and ribose moieties (signals 4 and 5, see Fig. S4B) and have relative saturation percentages around 35%. We analyzed two of the signals (8.7 and 8.2 ppm), and the increase in the peaks’ integrals with the increasing NADP^+^ concentration was also observed (Fig. 3B). In addition, NDH-2B was titrated with NADP^+^ in the presence of DMN and *vice versa* to investigate whether the two substrates were competing for the same binding site. The STD-NMR spectra of the titration of NDH-2B with DMN in the presence of NADP^+^ originated seven peaks, two from DMN and five from NADP^+^. The increase in DMN concentration had no relevant effect on the STD amplification factor of NADP^+^, indicating that DMN does not affect the binding of NADP^+^ to NDH-2B (Fig. 3C). In the case of the titration of NDH-2B with NADP^+^ in the presence of DMN, we observed that the STD amplification factor of NADP^+^ increased with the increasing of NADP^+^ in solution, while the STD ampliﬁcation factor of DMN did not change significantly (Fig. 3D). These results indicate that the binding of one substrate does not influence the binding of the other one, suggesting that the two substrates, NADPH and quinone, have different binding sites.

**Fig 4:**
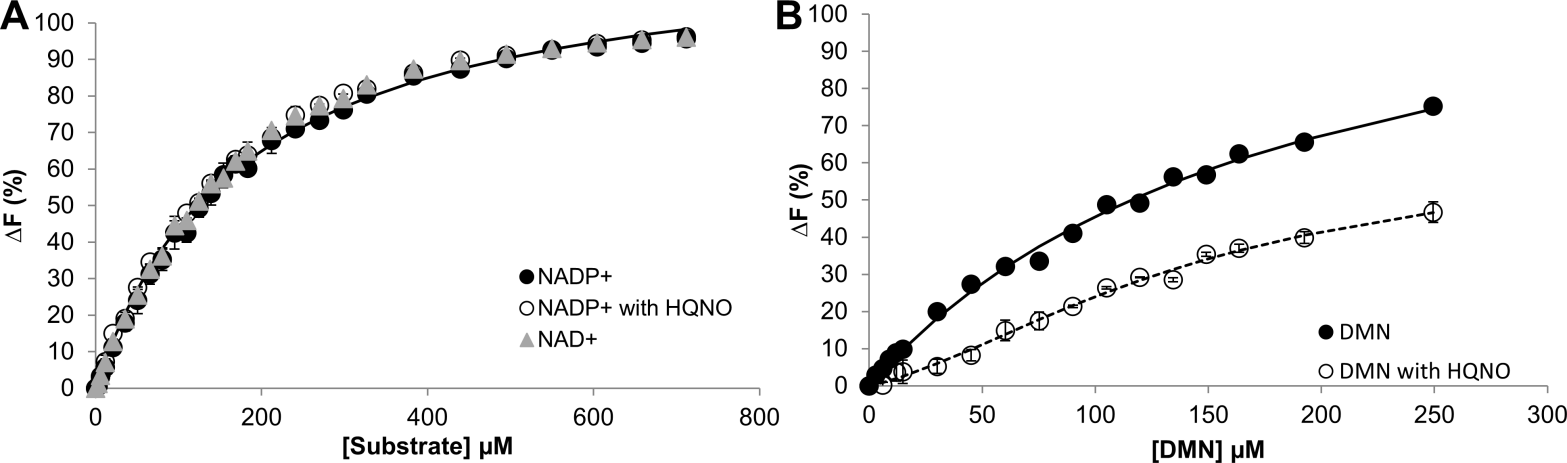
NDH-2B-substrate interaction monitored by fluorescence quenching titrations (**A**) Change in the ﬂuorescence emission at 350 nm with excitation at 280 nm of NDH-2B by sequential addition of NADP^+^ in the absence (closed circles) or presence of HQNO (open circles); sequential addition of NAD^+^ (triangles). Data were fitted with a hyperbolic equation. (**B**) Change in the ﬂuorescence emission at 350 nm with excitation at 295 nm of NDH-2B by sequential addition of DMN in the absence (closed circles) or presence of HQNO (open circles). Data were fitted with the Monod-Wyman-Changeux model equation. Two micromolar NDH-2B in 25 mM MES Bis Tris Propane, 250 mM NaCl pH 5.5 was used. Presented values are the median of at least three independent experiments; bars represent standard mean deviation.

Protein ligand interactions by NDH-2B were also investigated by fluorescence quenching titrations. NDH-2B has four tryptophan residues (W192, W228, W298, and W347). The fluorescence emission spectrum of NDH-2B with excitation at 280 nm exhibited the characteristics of a typical tryptophan ﬂuorescence spectrum with a broad maximum at around 340 nm, whereas the fluorescence emission spectrum with excitation at 450 nm was typical of a flavoprotein, presenting a band with a maximum intensity at 530 nm. Upon the addition of NADP^+^ or DMN, only slight changes were observed in the region of the flavin fluorescence emission spectra. By contrast, the intensity in the region of the tryptophan fluorescence emission spectra changed significantly upon the addition of each compound. The maximum change in tryptophan fluorescence observed upon titration with NADP^+^ and NAD^+^ is approximately 10 times larger than that observed with the DMN (data not shown), indicating that the environment of the tryptophan residues is perturbed differently by the quinone and by NADP^+^ or NAD^+^. The microscopic dissociation constants (*K*_*s*_) obtained for NADP^+^ and NAD^+^ were similar: 182 ± 29 µM for NADP^+^ and 170 ± 7.6 µM for NAD^+^ (Fig. 4A). When titrated independently with the quinone, DMN (Fig. 4B), a *K*_*s*_ of 160 ± 35 µM was obtained. NDH-2B was also titrated with HQNO, and the maximum change in tryptophan fluorescence was of the order of magnitude of that observed with DMN, which suggests a similar interaction of the quinone and the inhibitor. Moreover, titrations of NDH-2B with NADP^+^ and DMN in the presence of HQNO revealed that the profile of NDH-2B with NADP^+^ was similar to that observed in its absence, indicating that HQNO binds to a site that is distant from the NADP^+^ binding pocket. However, titration of the quinone, in the presence of HQNO, presented a different profile suggesting, as already observed for NDH-2A (9), that HQNO is affecting the binding of the quinone (Fig. 4A and B).

### Growth analysis of the knockout mutants reveals growth defect of the Δ*ndh-2a* mutant

To explore the cellular impact of each NDH-2 in *S. aureus, ndh-2a* and *ndh-2b* null mutants were constructed, in the background of *S. aureus* strain NCTC8325-4 by removing the entire corresponding gene from the chromosome and leaving no resistance marker.

**Fig 5:**
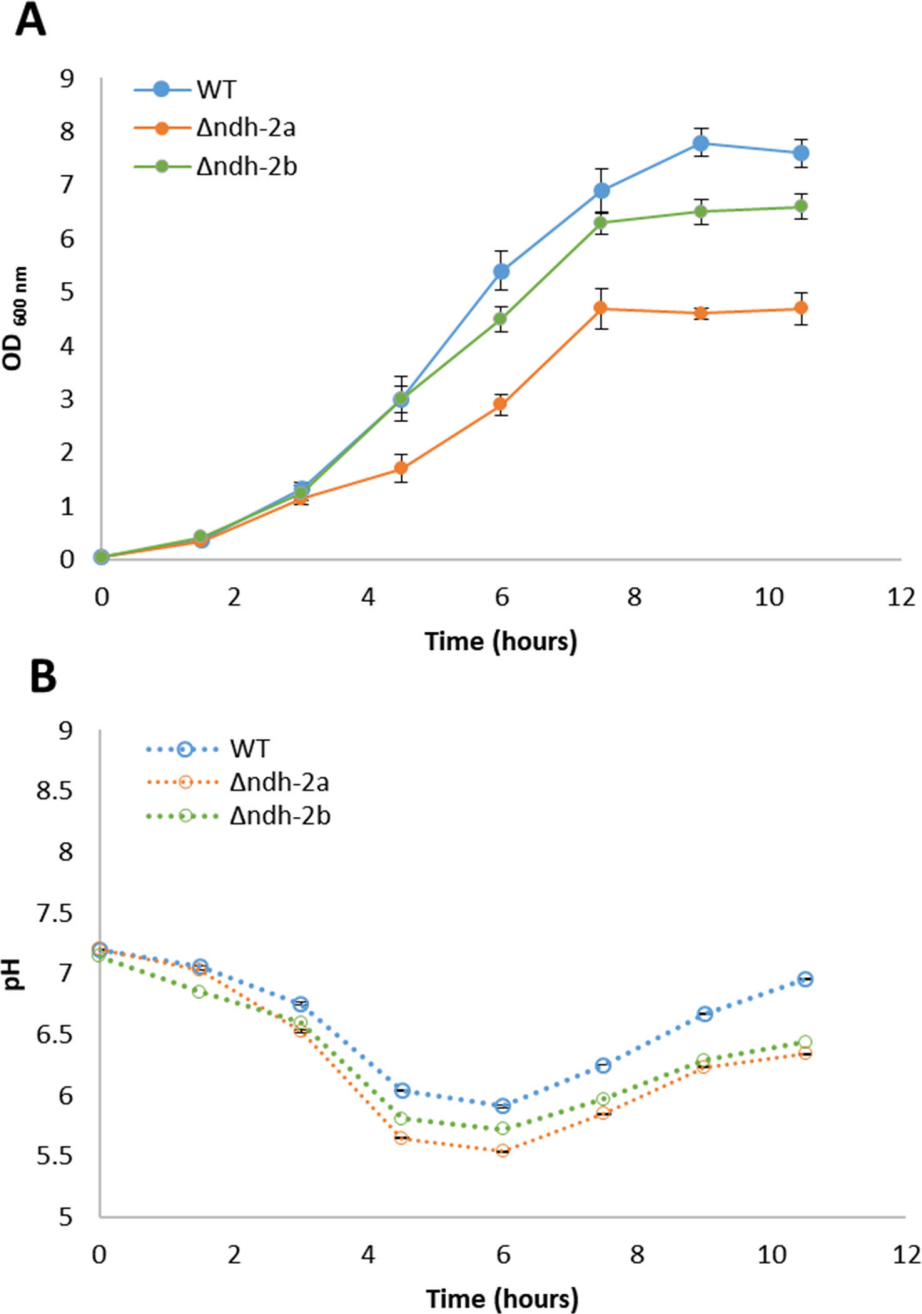
Growth of *S. aureus* wild-type strain NCTC8325-4, Δ*ndh-2a,* and Δ*ndh-2b* in TSB. The absorbance at 600 nm and the pH values were measured at 1.5-hour intervals. The results presented are representative of three independent experiments (respective error bars). Comparison of the growths of *S. aureus* wild-type (blue), ∆*ndh-2a* (orange), and ∆*ndh-2b* (green) strains by optical density (**A**) and pH (**B**).

*S. aureus* cell growth assessment of wild-type and mutant strains, Δ*ndh-2a* and Δ*ndh-2b*, was performed in a rich medium, tryptic soy broth (TSB), containing 14 mM glucose and was evaluated by monitoring optical density at 600 nm (OD_600_) and pH changes. The results presented in Fig. 5A show different growths of the wild-type strain and the Δ*ndh-2a* strain, this last one reaching a maximal OD_600_ approximately of 5. The other mutant strain (Δ*ndh-2b*) had a more similar growth behavior to the wild-type strain with a slight delay (Fig. 5A). The pH profile, observed for all strains, is characterized by a continuous decrease from the beginning of the growth until ~6 hours, followed by an increase in pH (Fig. 5B).

### Cell volume and cell cycle of Δ*ndh-2a* and Δ*ndh-2b* mutant strains are altered compared to the WT strain

**Fig 6:**
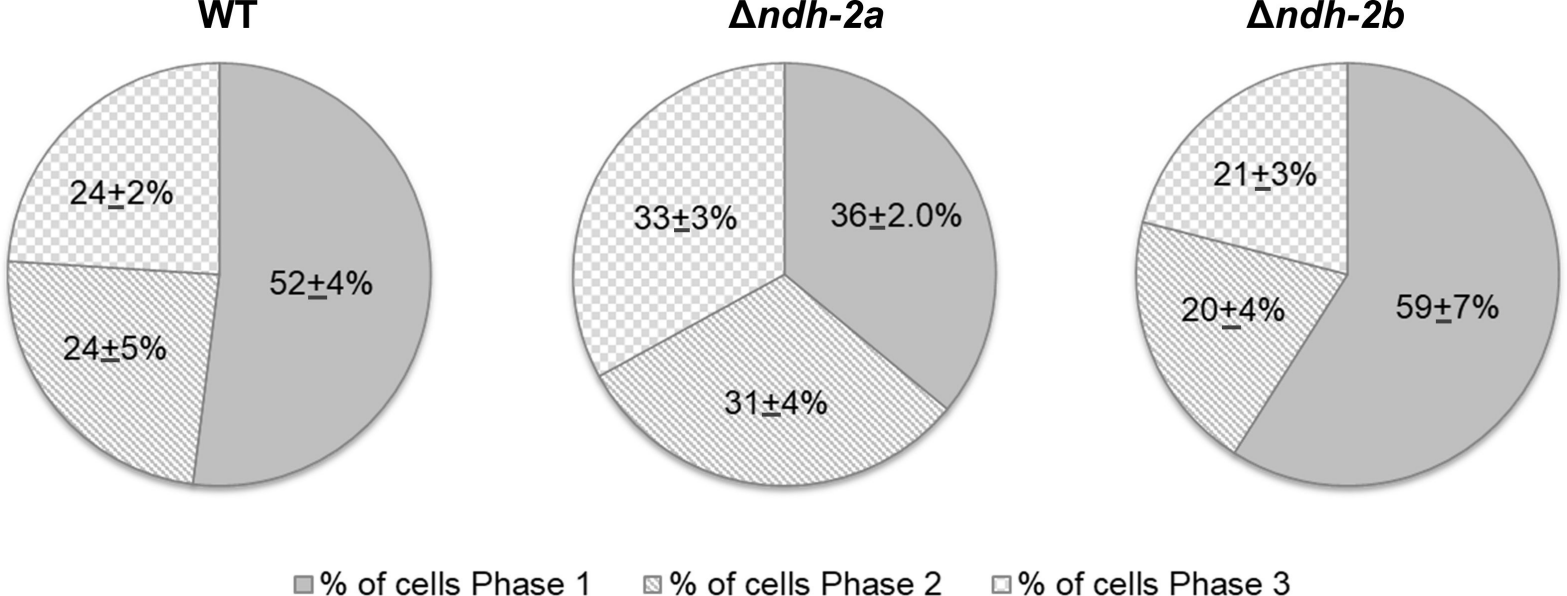
Distribution of cell cycle phases of *S. aureus* NCTC8325-4 (WT) and mutants Δ*ndh-2a* and Δ*ndh-2b*. Frequency of cells in each phase of the cell cycle calculated from SR-SIM images. Δ*ndh-2a* strain has shorter Phase 1 and/or longer Phases 2 and 3, indicating altered cell cycle progression (*n* = 600 cells per strain). The results presented are representative of at least three independent experiments.

Since the absence of NDH-2A or NDH-2B impacts growth rates, the mutants’ cell cycle progression could also be altered. The *S. aureus* cell cycle was described as presenting three phases: in Phase 1 (P1), cells have recently divided and have not initiated the synthesis of the septum; in Phase 2 (P2), cells are undergoing septum synthesis; and in Phase 3 (P3), cells have a complete septum and are going to split into two daughter cells (23). The obtained results showed that the wild-type and ∆*ndh-2b* strains presented approximately more than half of the cells in Phase 1 of the cell cycle, and the other half of the cells were almost equally distributed between Phase 2 and Phase 3. In contrast, the ∆*ndh-2a* mutant presented a similar percentage of cells in the three phases of the cell cycle, which indicates that cell cycle progression in this mutant is modified (Fig. 6).

**Fig 7:**
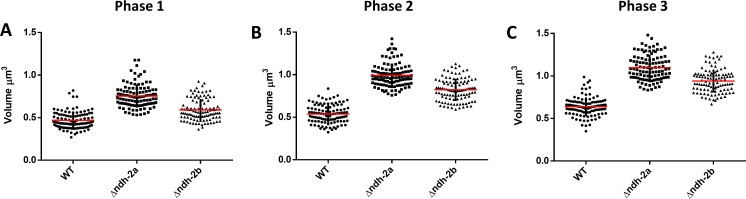
Morphological changes during the *S. aureus* cell cycle. Cell volume was measured at each phase of the cell cycle of the *S. aureus* NCTC8325-4 (WT, circles), Δ*ndh-2a* (squares), and Δ*ndh-2b* (triangles) cells in Phase 1 (**A**), Phase 2 (**B**), and Phase 3 (**C**). Cells increased their volume continuously throughout the cell cycle, from an average volume of 0.47 ± 0.1 µm^3^ at P1 to an average volume of 0.64 ± 0.11 µm^3^ at P3 in the case of the WT strain (*n* = 105 cells for each phase). Scatter plots showing cell size with the median of each distribution indicated by a red line. Bars represent the mean standard deviation. See Table S1 for more details.

To assess the impact of the absence of each NDH-2 on *S. aureus* morphology dynamics during the cell cycle, the cell volumes (*n* = 105 cells for each phase) at each phase (P1, P2, and P3) of the *S. aureus* cell cycle were determined. The mutants presented larger cell volumes than the WT for all phases, with *∆ndh-2a* exhibiting the largest volumes (Table S1; Fig. 7).

### Δ*ndh-2a* strain excretes high levels of lactate

**Fig 8:**
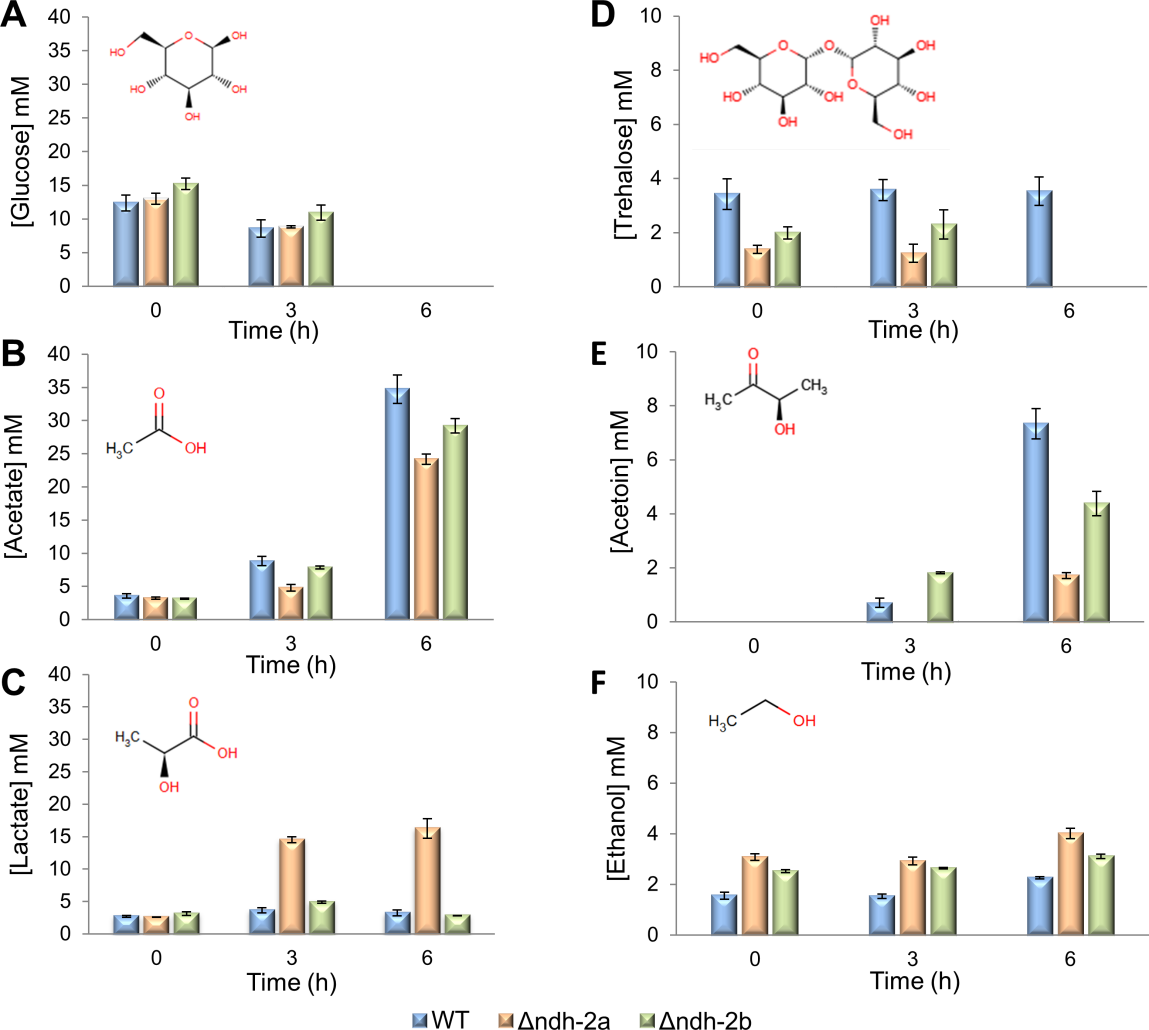
Quantification of the main extracellular metabolites. Glucose (**A**), acetate (**B**), lactate (**C**), trehalose (**D**), acetoin (**E**), and ethanol (**F**). *S. aureus* wild-type (blue), Δ*ndh-2a* (orange), and Δ*ndh-2b* (green) strains were grown in TSB under aerobic conditions, and the glucose, acetate, lactate, trehalose, acetoin, and ethanol concentrations in the culture supernatants were monitored by ^1^H-NMR. The results are the median of at least three independent experiments, and bars represent the standard mean distribution.

The phenotypes observed in cell growth, size, and cycle progression were hypothesized to be due to alterations in the energy metabolism in which NDH-2s intervene. To investigate the impact of each NDH-2 in the energy metabolism of *S. aureus*, thorough NMR-based metabolomics analyses were performed, obtaining a partial, time-resolved concentration profile of the extracellular metabolites (Fig. S5). When inspecting the general profile, the difference in the concentration of lactate was immediately noticed, and a closer inspection shows further differences, specifically in the concentrations of acetoin (Fig. 8; Fig. S5).

The wild-type NCTC8325-4, ∆*ndh-2a,* and ∆*ndh-2b* strains catabolized, in 3–6 hours, all the glucose (approximately 14 mM) present in the medium at the beginning of the growth experiments (Fig. 8). *S. aureus* was reported to catabolize glucose by the glycolytic and pentose phosphate pathways (1). Moreover, *S. aureus* metabolism is regulated by a carbon catabolite repressor process under which the citric acid cycle is repressed in the presence of highly reduced carbohydrates such as glucose (24). Thus, in this situation, glucose is oxidized to pyruvate, which is further oxidized to acetate, if NADH produced in the glycolytic pathway can be reoxidized by the respiratory chain. In fact, it can be observed that along with glucose consumption, acetate was produced by all the strains by 6 hours of growth (Fig. 8B). At this time, ~35 mM acetate was produced by the wild-type strain, ~29 mM by the Δ*ndh-2b* mutant, and ~24 mM by the ∆*ndh-2a* mutant. Moreover, accumulation of acetate in the culture medium is probably responsible for the characteristic pH decrease observed in all growth curves (Fig. 5B).

Usually, along with acetate, acetoin is produced to allow cells to control internal acidification (25). Acetoin is obtained from two molecules of pyruvate, via 2-acetolactate. By 6 hours of growth, ~7.5 mM acetoin was produced by the wild-type strain, ~1.7 mM by the Δ*ndh-2a* mutant, and ~4.4 mM by the ∆*ndh-2b* mutant (Fig. 8E).

Lactate was produced by the three strains, although after 6 hours of growth, the WT and ∆*ndh-2b* strains produced much lower amounts of this metabolite (~3.3 and ~2.9 mM lactate in WT and ∆*ndh-2b,* respectively) than the ∆*ndh-2a* strain, which accumulates approximately 16.2 mM (almost five times higher) (Fig. 8B and C). Most interestingly, at 3 hours of growth, the ∆*ndh-2a* strain has already accumulated ~14.5 mM lactate, a much higher value than that of acetate (~4.8 mM). However, at 6 hours of growth of the ∆*ndh-2a* strain, the lactate concentration increased only slightly (1.7 mM), while acetate concentration increased notably (19.5 mM) (Fig. 8C).

Ethanol was also identified among the extracellular metabolites, with ∆*ndh-2a* presenting slightly increased concentrations of this metabolite (4 mM) when compared to the other strains (2 mM by WT and 3 mM by ∆*ndh-2b*, at the 6-hour mark) (Fig. 8F).

The observed profile of the several metabolites of the ∆*ndh-2b* mutant does not present pronounced differences from that of the wild-type strain. Nevertheless, this mutant grows slower than wild-type NCTC8325-4, suggesting that there may be energy restrictions, an idea supported by the observation that the ∆*ndh-2b* mutant, similarly to ∆*ndh-2a*, exhausted the available trehalose by 6 hours of growth (Fig. 8D). A difference in the production of acetoin was also detected, by 3 hours of growth, by ∆*ndh-2b* mutant in relation to the other strains. This mutant started producing acetoin as the wild-type strain, but by 3 hours of growth, it accumulated more than double of acetoin than the wild type (1.8 vs 0.7 mM) (Fig. 8E).

Some amino acids such as alanine, aspartate, and glycine could also be identified (Fig. S3). Alanine and aspartate were not consumed by any of the strains under study, but consumption of glycine by all three strains in a similar way was observed.

### *Δndh-2a* shows the least efficient conversion of nutrients into biomass

**Fig 9:**
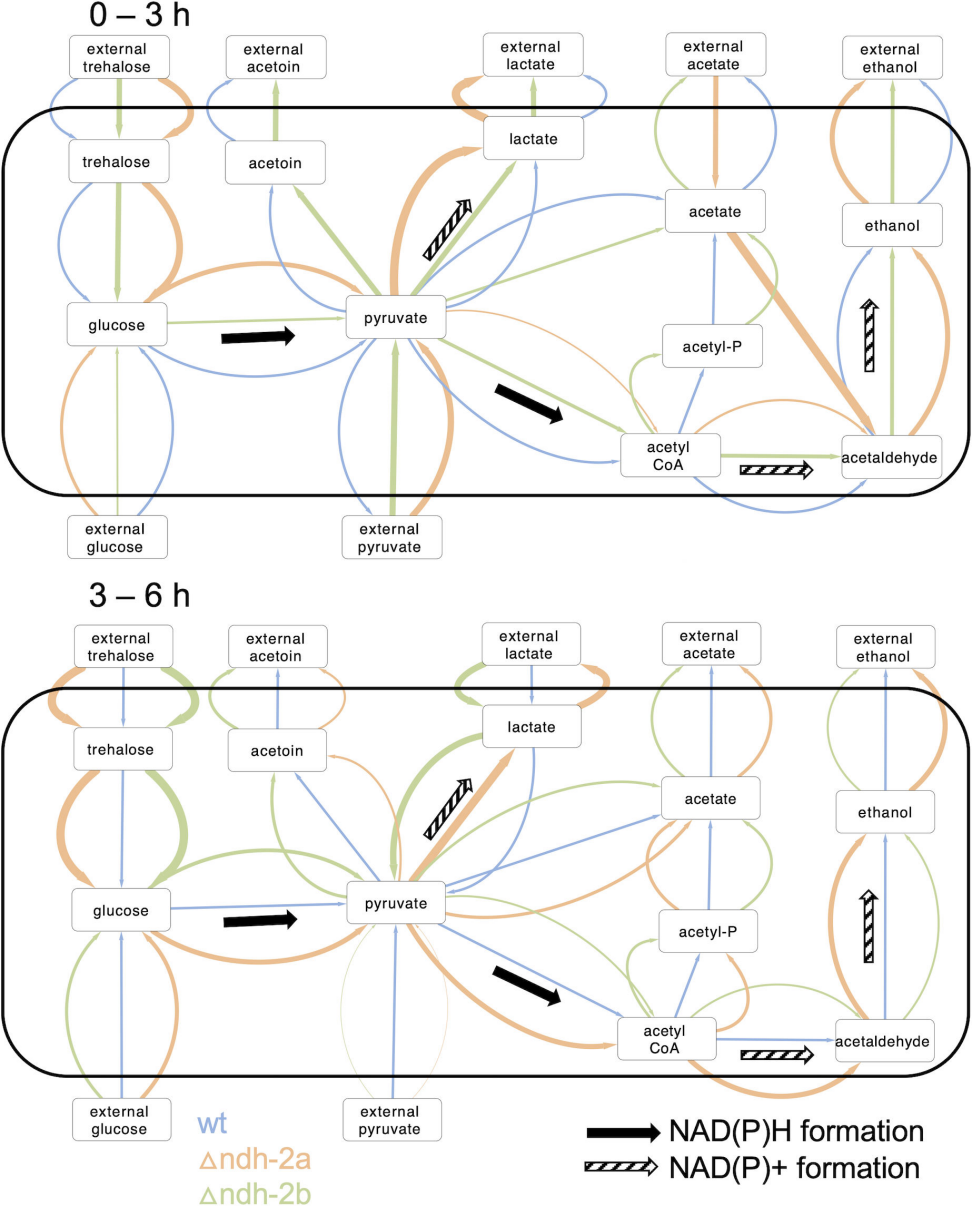
Metabolic flux distribution of *S. aureus* wild-type NCTC8325-4 and Δ*ndh-2a* and Δ*ndh-2b* mutants. Metabolic flux distributions for 0–3 hours growth period (top) and 3–6 hours growth period (bottom). The different fluxes are represented by arrows: blue for the wild-type strain, orange for the Δ*ndh-2a* mutant, and green for the Δ*ndh-2b* mutant. The metabolic flux of each metabolite is represented relatively to the same flux in the wild-type strain and is reflected by each arrow thickness. All wild-type strain fluxes’ arrows (blue) represent one unit of thickness.

In order to further understand the metabolomic observations, a metabolomic flux distribution analysis based on the NMR data was performed. In this analysis, the measurements of external metabolites were used to quantify the rate at which these are entering or leaving the cell (Table S2). In the metabolic network, the most direct metabolic pathways that connect the external metabolites were identified, and the minimal flux through those pathways that is compatible with the observed influxes and effluxes was estimated. For this estimation, it was considered that trehalose and glucose are completely converted to pyruvate. The fluxes were estimated in relation to pyruvate (in pyruvate equivalents); therefore, glucose and trehalose-consuming fluxes were doubled and quadruplicated, respectively, because each molecule of glucose originates two molecules of pyruvate and each molecule of trehalose originates four molecules of pyruvate. It was also considered that lactate is produced from one molecule of pyruvate and acetoin is produced from two molecules of pyruvate, via 2-acetolactate, and therefore, the acetoin production flux was doubled. Ethanol is produced from acetate or pyruvate (via acetyl-CoA). The metabolomic flux distributions obtained for the three strains show that, in the first 3 hours of growth, the ∆*ndh-2a* mutant presents the highest influxes of glucose and trehalose (Fig. 9; Table S2). Moreover, it is also the only strain that shows an influx of acetate, while the other two strains present effluxes of this metabolite. In addition, ∆*ndh-2a* has the highest effluxes of lactate and ethanol and shows no flux from pyruvate to acetate. It is also the only strain displaying a flux from acetate toward the formation of acetaldehyde (Fig. 9; Table S2). In the 3–6 hours growth period, the ∆*ndh-2a* is still the strain presenting the highest influxes of glucose and trehalose and still shows an efflux of lactate, but now these fluxes are accompanied by an efflux of acetate (instead of an influx as observed from 0 to 3 hours). Curiously, this acetate efflux is the highest observed for the three strains in this period. Moreover, a flux from pyruvate to acetate can be now estimated (Fig. 9; Table S2).

**Fig 10:**
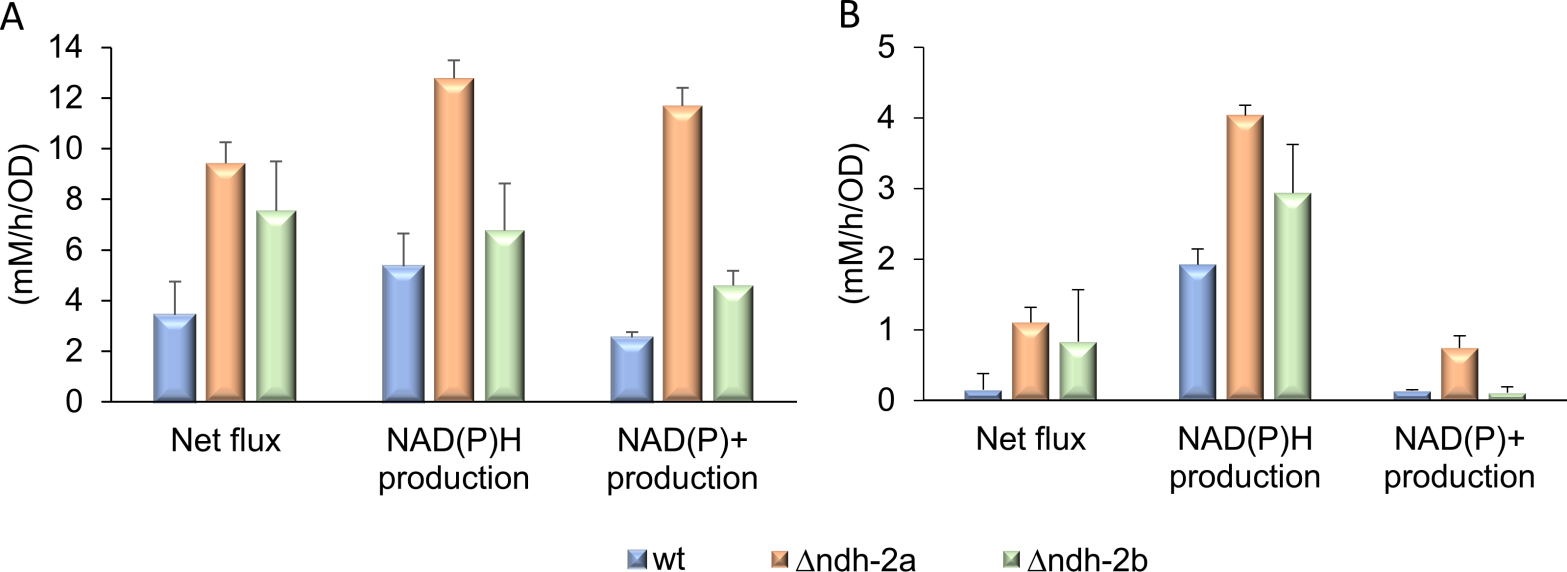
Net flux of metabolites and NADH and NAD^+^ production by *S. aureus* (mM/h/OD) in the time intervals from 0 to 3 hours (**A**) and 3 to 6 hours (**B**). *S. aureus* wild-type (blue), Δ*ndh-2a* (orange), and Δ*ndh-2b* (green) mutants. Line bars represent mean standard deviations.

The exchange fluxes also allow to estimate the net influx of nutrients into the cells (using three carbon atoms as a reference unit). Globally, in the 0–3 hours growth period, ∆*ndh-2a* strain is the one with the highest net flux of metabolites, followed by ∆*ndh-2b* (Fig. 10; Table S3). In the time interval from 3 to 6 hours, all strains grew slower than between 0 and 3 hours, had lower nutrient influxes, and again the wild-type strain presented the lowest net metabolite flux, while ∆*ndh-2a* was the strain showing the highest metabolite flux (Fig. 10; Table S3).

Since Δ*ndh-2a* and Δ*ndh-2b* lack genes encoding NAD(P)H:quinone oxidoreductases, the production and consumption rates of NAD(P)H were analyzed (Fig. 10; Table S4). For this analysis, it was considered that for each pyruvate produced (from trehalose or glucose), one NAD^+^ is converted to NADH, each lactate produced from pyruvate converts one NADH to NAD^+^, production of ethanol from acetate converts two molecules of NADH to NAD^+^, and production of ethanol from pyruvate converts 1.5 molecules of NADH to NAD^+^ (one NADH may be obtained from the conversion of pyruvate to acetyl-CoA, but alternatively, the conversion of pyruvate to formate does not generate NADH, so 0.5 NADH formation was estimated, and one NADH is consumed from acetyl-CoA to acetaldehyde and another is consumed from acetaldehyde to ethanol). Using these considerations, the net production of NADH by glycolysis and the production of acetate, acetoin, lactate, and ethanol can be estimated. These estimates are equal to or higher than the real production rates because (i) glycolysis intermediates can be used for alternative pathways and are neither converted to pyruvate nor lead to the production of NADH; and (ii) excess pyruvate can generate extra NAD^+^ if converted to malate or alanine, for example. During the 0–3-hour time interval, *∆ndh-2a* strain both produces and consumes NAD(P)H the most (as shown in Table S4). This results in the least net NAD(P)H production, making *∆ndh-2a* the least efficient strain when considering net NAD(P)H production per net metabolite flux (Fig. 10; Table S4).

### Pyruvate metabolism is strongly affected in Δ*ndh-2a* and Δ*ndh-2b* mutants

**Fig 11:**
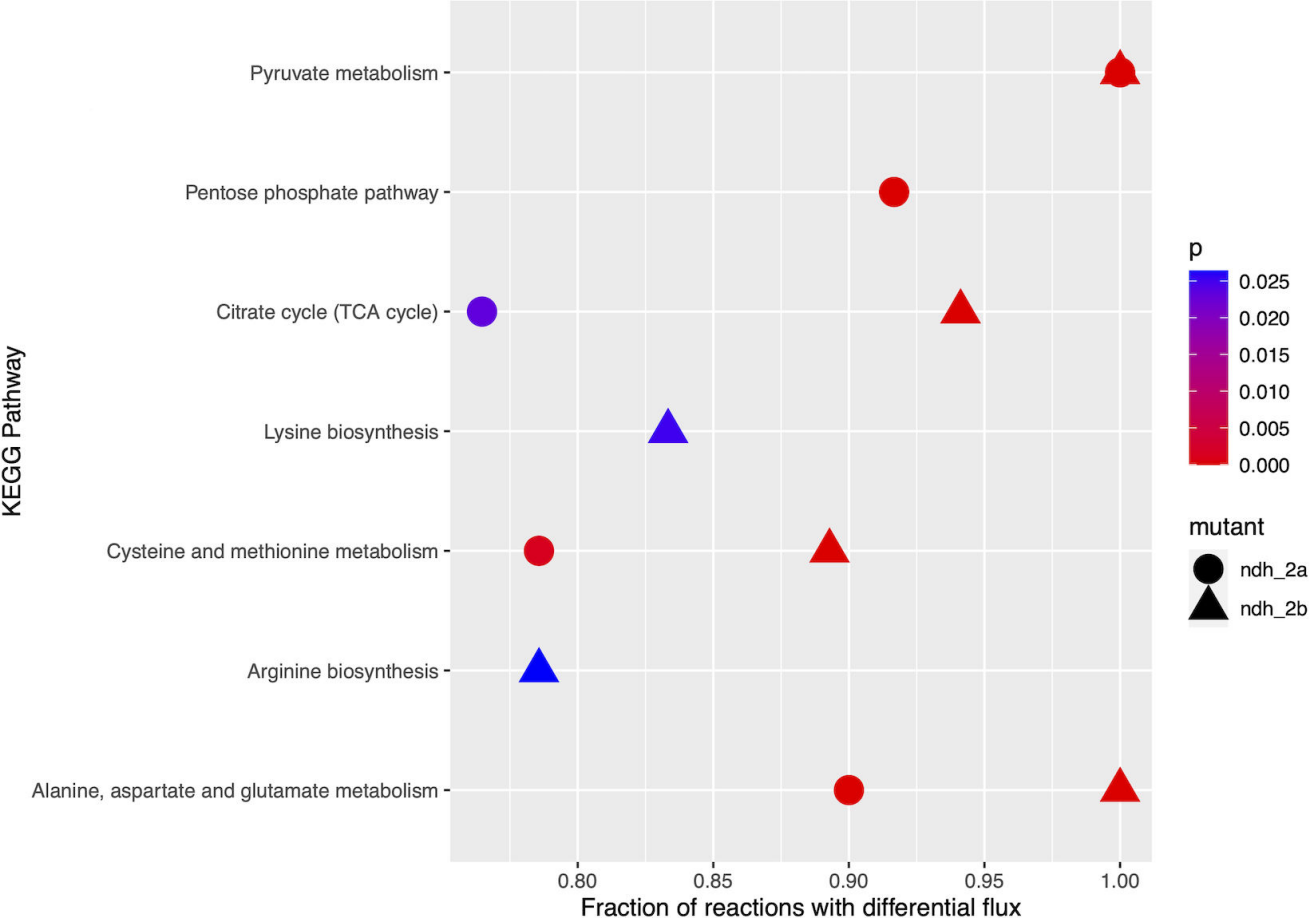
Network diffusion algorithm. Pathways most affected by the lack of Ndh-2A (circles) or Ndh-2B (triangles), in which at least one-third of the involved metabolites were part of shortest paths linking these to the monitored external metabolites. The *P*-value was the fraction of the random sets that showed an equal or higher number of associated reactions. Pathways with a *P*-value lower than 0.03 were considered as significantly enriched. Reactions included in each pathway were the ones defined in KEGG.

To detect which parts of the metabolic network are most affected by the lack of Ndh-2A or Ndh-2B, the exchange fluxes (from the 0–3-hour period) were considered as inputs for a network diffusion algorithm, using the KEGG pathways. Each reaction in this network was scored according to the diffusion received from both the source and sink external metabolites. Figure 11 indicates KEGG pathways for each mutant that has reactions with higher diffusion scores than the wild type and which were selected from the top half of total reactions with significant differences in diffusion scores (Table S5). Only pathways with a number of reactions between 10 and 30 and a significant difference from the expected proportion of reactions in the top half in at least one of the mutants were considered. Ten and eight pathways are significantly affected in ∆*ndh-2a* and ∆*ndh-2b* mutants, respectively (Fig. 11; Table S5), with pyruvate metabolism strongly affected by both mutations. Interestingly, the pentose phosphate pathway seemed to be highly affected in ∆*ndh-2a* but not in ∆*ndh-2b* (Fig. 11), in which the number of altered reactions is significantly lower than expected by chance. Also, the citric acid cycle seems to be the most influenced by the *ndh-2b* deletion, although differences are also observed in the case of the *ndh-2a* deletion, and as a possible consequence, some amino acids’ metabolisms are also altered by the deletions (Fig. 11).

## DISCUSSION

In this work, we conducted a comprehensive biochemical characterization of NDH-2B, a NADPH:quinone oxidoreductase exhibiting maximal activity at pH 5.5. This contrasts with the previously characterized NDH-2A, which, in our current investigation, showed a preference for NADH oxidation over NADPH and reached maximal activity at approximately pH 7. Furthermore, when NDH-2B was reduced with NADPH, we did not observe the formation of a CT complex, unlike the case with NDH-2A when reduced by NADH. Our fast kinetic experiments revealed that, for NDH-2B, the rate-limiting step corresponds to its reduction by NADPH. This contrasts with what was observed for NDH-2A, in which the oxidation by the quinone was identified as the rate-limiting step. In this case, we observed that the CT complex slowed down the quinone reduction, and we suggested that this may contribute to maintaining a low quinol/quinone ratio, thereby preventing the production of reactive oxygen species through unspecific quinol oxidation (9, 21). For NDH-2B, the reduction of the flavin is inherently slow, making the establishment of a charge transfer complex between NADP^+^ and FADH_2_ to slow down quinone reduction and, consequently, prevent the overproduction of reactive oxygen species, potentially unnecessary. Additionally, our study, involving STD-NMR measurements and fluorescence quenching titrations, indicates that the two substrates, NADPH and quinone, have different binding sites. HQNO, in particular, binds to a site separate from that of NADP^+^, only interfering with quinone binding, as observed for NDH-2A (9).

To address the question of whether the different *in vitro* activities translated into different cellular functions, we investigated these enzymes through the design of knockout mutants (∆*ndh-2a* and ∆*ndh-2b*). ∆*ndh-2a* mutant grows slower than ∆*ndh-2b* or the wild-type strain NCTC8325-4, reaching the lowest OD_600_ value in the stationary phase. This result agrees with the published study of NDH-2s from *S. aureus* that showed that loss of NDH-2A (ndhC, SAB0807 *S. aureus* RF122 strain) strongly impaired staphylococcal growth when compared to wild-type bacteria (7). We also analyzed how the cell cycle, which includes septum initiation (Phase 1), synthesis (Phase 2), and splitting (Phase 3), progressed in the WT and in the mutants by assessing the number of cells in each cell cycle phase. ∆*ndh-2a* had a higher number of cells in Phase 2 and Phase 3 of the cell cycle, indicating that normal cell cycle progression is compromised in this mutant. Furthermore, we assessed the impact of the absence of these proteins at the single-cell level by measuring the volume of cells from the ∆*ndh-2a* and ∆*ndh-2b* mutants. Both mutants exhibited an increase in cell volume compared to wild-type cells across the cell cycle, suggesting that cell division may have been altered, as increased cell volume is a characteristic feature of cell division mutants (26–30). In this way, we conclude that both cell size control and cell cycle progression have been compromised in the mutants, likely due to alterations in their respective energy metabolisms.

**Fig 12:**
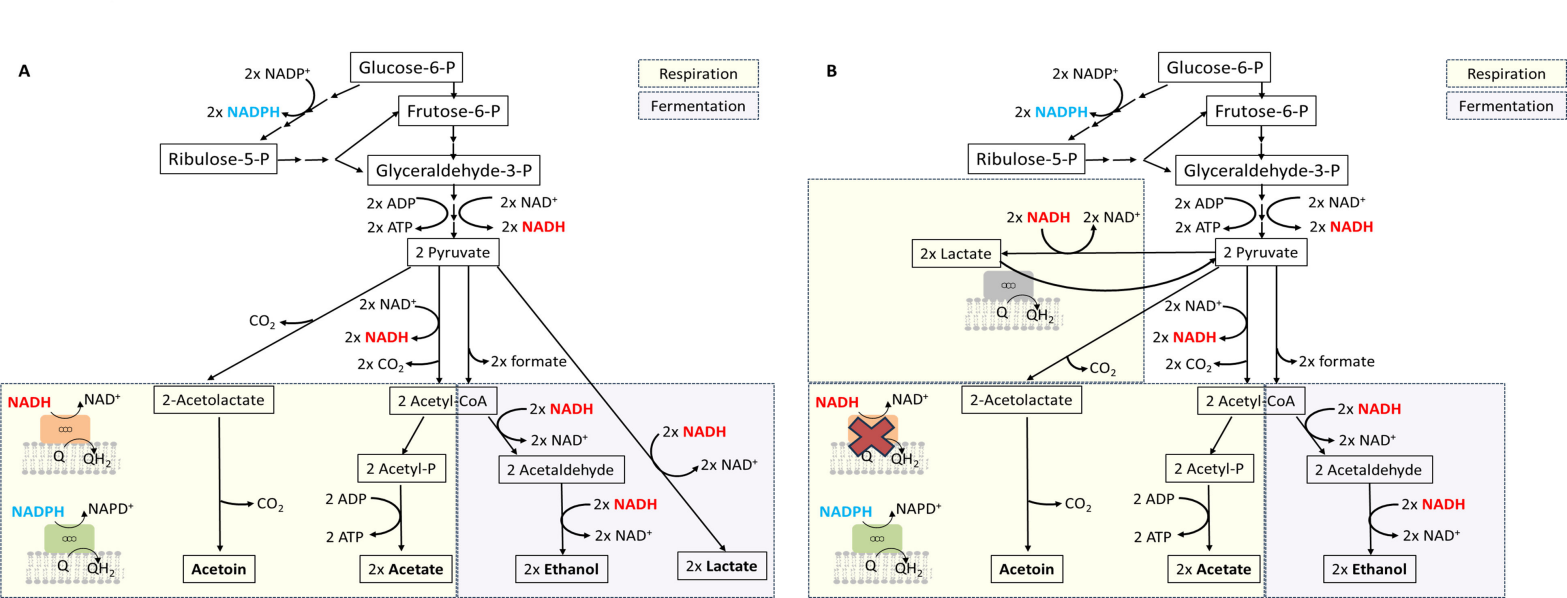
Schematic representation of the central carbon metabolism of *S. aureus*. Production of NADH (red), NADPH (blue), and ATP is indicated. (A) Pathways operating in the wild type and (B) pathways operating in the absence of NADH:quinone oxidoreductase, NDH-2A, showing the proposed change in lactate metabolism.

We examined the energy metabolism of the three strains (WT, ∆*ndh-2a*, and ∆*ndh-2b*) by scrutinizing their extracellular metabolites through NMR analyses and metabolomic flux studies. The extracellular accumulation of acetate indicates that *S. aureus* is respiring. This is because NADH, produced in the glycolytic pathway, can only be regenerated to NAD^+^ by the respiratory chain as no pathway leading to acetate oxidizes NADH (Fig. 12). In instances in which NADH, generated in the glycolytic pathway, cannot be reoxidized by the respiratory chain—potentially due to enzymatic impairment or oxygen limitation—it can be reoxidized via the reduction of pyruvate to lactate or to acetaldehyde, and subsequently to ethanol. In this scenario, the NAD^+^ required to sustain cellular metabolism becomes available (Fig. 12).

Interestingly, the *∆ndh-2a* strain, in the 0–3-hour growth interval, accumulates a much higher quantity of lactate than acetate. This implies that NAD^+^ regeneration occurs primarily through NADH:pyruvate oxidoreductase and not by the respiratory chain—a finding consistent with the absence of NDH-2A in this mutant. These data suggest the adoption of fermentative metabolism rather than a respiratory one, resulting in reduced energy yield and subsequently observed diminished cell growth. Moreover, our metabolomic flux studies indicate that ∆*ndh-2a* mutant is the one that presents the highest net flux of metabolites, followed by ∆*ndh-2b* mutant. Knowing that the wild type is the strain with the fastest growth and ∆*ndh-2a* is the strain with the slowest growth, it is reasonable to infer that the mutants experience a less efficient conversion of nutrients into biomass. In this way, the absence of NDH-2A and NDH-2B impacts the biomass conversion efficiency differently, with the Δ*ndh-2a* mutant facing a more pronounced challenge than the Δ*ndh-2b* mutant. The compromised energy metabolism in the mutants, particularly evident in *∆ndh-2a*, may account for the observed effects on cell volume and cell cycle. As energy is pivotal for cell division, the *∆ndh-2a* mutant may experience constraints in its cell cycle due to limited energy availability compared to the wild-type.

By 6 hours of growth, we observed that ∆*ndh-2a* mutant still accumulates lactate, albeit to a lesser extent, and intriguingly begins to accumulate acetate. This observation suggests a potential replacement of the NDH-2A function during this time frame. One plausible explanation, supported by the metabolomic flux analyses, is that, by this time, cells start to import the previously produced lactate, which is a substrate of lactate:quinone oxidoreductase, to produce pyruvate and quinol (Fig. 12B). Pyruvate is subsequently metabolized to produce acetate, using the same pathways as the wild-type strain. This hypothesis explains acetate accumulation by 6 hours of growth and the shift back to respiratory metabolism. At this time, a steady state in lactate levels would have been reached between its production by NADH:pyruvate oxidoreductase, which produces NAD^+^, and its reoxidation back to pyruvate that produces quinol, which is oxidized by the respiratory chain. In this way, this cycle would constitute an indirect way of oxidizing NADH by the quinones and consequently by the respiratory chain (31). Altogether, the data indicate that, in the absence of NDH-2A, cells employ a fermentation process to regenerate NAD^+^, which produces lactate and when the amount of lactate reaches a certain threshold, cells are able to indirectly reoxidize NADH by the respiratory chain via NADH:pyruvate oxidoreductase and lactate:quinone oxidoreductase (Fig. 12B). Nevertheless, this seems to be an energy-constrained situation because we also observed that ∆*ndh-2a* mutant, but not the wild-type strain, exhausted the available trehalose (probably used as an energy source) by 6 hours of growth.

The *∆ndh-2b* mutant strain exhibited a relatively minor metabolic phenotype compared to the wild type, primarily characterized by an earlier accumulation of acetoin. Acetoin is derived from two molecules of pyruvate through 2-acetolactate, a pathway not involving the regeneration of NAD^+^. Therefore, the accumulation of acetoin also serves as an indicator that the cells perform a respiratory metabolism (Fig. 12). It is worth noting that acetoin production has been demonstrated to enable cells to regulate internal acidification (25), and this may indicate that ∆*ndh-2b* mutant needs to compensate internal pH earlier than the wild type. The reason for this need remains to be identified.

Intriguingly, our metabolomic flux analyses reveal a significant impact on the pentose phosphate pathway in ∆*ndh-2a* but not in ∆*ndh-2b*. This observation may suggest that the ∆*ndh-2a* mutant attempted to utilize this pathway as an alternative to glycolysis for the production of NADPH instead of NADH (Fig. 12), which would be advantageous in a scenario in which NADH:quinone oxidoreductase is absent and NADPH:quinone oxidoreductase is present. However, the pentose phosphate pathway converges to the glycolytic pathway and does not circumvent the total production of NADH.

Here, we investigated NDH-2B and demonstrated that it functions as a NADPH:quinone oxidoreductase, indicating a catalytic function distinct from that of NDH-2A. We observed that NDH-2B does not establish a charge-transfer complex in the presence of NADPH, and its reduction by this substrate is the catalytic rate-limiting step. Additionally, we explored the cellular roles of the two NDH-2s and observed that the lack of NDH-2A or NDH-2B impacts cell growth, volume, and division differently. Taken together, the NDH-2s knockouts offer a unique opportunity to investigate how *S. aureus* adapts and modifies its metabolism in response to imposed constraints. Notably, the losses of NDH-2A and NDH-2B result in different phenotypes, emphasizing that each enzyme has specific cellular roles in *S. aureus* metabolism.

The knowledge of the metabolic adaptation strategies of *S. aureus* to environmental and bioenergetics challenges can provide clues to therapeutic strategies that interfere with the ability of the pathogen to successfully adapt when it invades different niches within its host.

## MATERIALS AND METHODS

### NDH-2B expression, purification, and biochemical characterization

The gene SAOUHSC_00875, coding for NDH-2B from *Staphylococcus aureus* strain NCTC8325-4, was cloned into pET-28a(+) (Novagen) and expressed in *Escherichia coli* Rosetta 2 (DE3) pLysS cells (Novagen). NDH-2B was purified in a similar procedure to that used for NDH-2A (SAOUHSC_00878), as described in Rosário et al. (20). Briefly, NDH-2B was purified from the membrane fraction by washing it with a buffer solution containing 2 M NaCl and purified through two chromatographic steps, a Q-sepharose high performance and size exclusion (S200) columns. The purified protein was analyzed by mass spectrometry at the MS Unit, ITQB/IBET (using a positive reflector MS and MS/MS modes in a 4800 plus MALDI-TOF/TOF mass spectrometer and the 4000 Series Explorer Software v.3.5.3) and stored at −80°C until needed.

Protein concentration was determined using the BCA Protein Assay Reagent (Pierce) and BSA (Thermo Fisher Scientific) as a standard. Protein purity was evaluated by SDS-PAGE. The flavin prosthetic group was identified by reverse-phase chromatography. The protein was denatured by incubation at 100°C for 10 min and removed by centrifugation. The supernatant was injected in a Waters, Nova Pack C18 60 Å 4 µm (3.9 × 150 mm) column operated in a Waters-Alliance HPLC system. The column was equilibrated with 100 mM ammonium acetate at pH 6 and 5% methanol (vol/vol), and the sample was eluted in the same buffer with a linear gradient from 5% to 15% methanol (vol/vol) at 1 mL min^−1^. Commercial FAD (785.55 Da, Merck) and FMN (456.34 Da, Sigma-Aldrich) were used as standards. The flavin content of the purified protein was determined spectroscopically in a Shimadzu UV-1603 spectrophotometer, using the extinction coefficient of 11.3 mM^−1^ cm^−1^ at 450 nm for the free oxidized flavin. The extraction of the flavin was done by incubating the protein in the presence of 10% trichloroacetic acid (vol/vol) for 10 min followed by centrifugation for 15 min.

To investigate the oligomerization state of the protein, a blue native-PAGE gel electrophoresis was carried out using a Novex Bis-Tris gel system (Invitrogen) according to the manufacturer’s specifications. Pre-cast NativePAGE Novex 4%–16% (vol/vol) Bis-Tris gel was run at pH 7, 150 V, and 4°C for 1 hour.

Thermal denaturation assays (25°C–90°C) were performed using a Peltier temperature controller with a rate of 0.5°C min^−1^, and the data were recorded in intervals of 0.5°C with an acquisition time of 0.1 min. Denaturation was monitored by fluorescence spectroscopy using excitation at 450 nm and emission at 530 nm to monitor flavin fluorescence.

UV-visible absorption spectroscopy was performed in an anaerobic chamber, and 0.35 mg of NDH-2B was reduced by the addition of NADPH (Panreac AppliChemand) (1:1) and oxidized by 2,3-dimethyl-1,4-naphthoquinone (1:1). DMN was synthesized from menadione (Sigma-Aldrich) according to Kruber (32). When needed, a mixture of 5 mM glucose (Roth), 4 U mL^−1^ glucose oxidase (Sigma-Aldrich), and 130 U mL^−1^ catalase (Sigma-Aldrich) was used to ensure total O_2_-free conditions.

NDH-2 reduction potential was determined by cyclic voltammetry using a silver chloride (Ag/AgCl) electrode as the reference, graphite (Pg) as the working electrode, platinum (Pt) as the counter electrode, and 100 mM potassium phosphate, pH 7.0 as the electrolyte solution. Measurements were done with scan rates of 20, 50, 100, 200, or 500 mV s^−1^, and data were then plotted as current (i) vs potential (E) to give the cyclic voltammogram trace.

### Steady-state kinetics

Enzyme activity assays were performed on a Shimadzu UV-1800 spectrophotometer monitoring the change in absorbance of the electron donor, NADPH or NADH (Sigma-Aldrich), at 340 nm. The activity pH profile study was performed with 0.2 µM NDH-2A or NDH-2B at 35°C and between pH 5.5 and 8 in 50 mM MES Bis Tris Propane, 250 mM NaCl. The activity assays were made inside an anaerobic chamber at 35°C, using DMN as an electron acceptor. The NADH or NADPH extinction coefficients of 6.22 mM^−1^ cm^−1^ were used to calculate the enzymes’ specific activity (µmol·min^−1^·mg protein^−1^). To determine the catalytic parameters for NDH-2B, activity assays were performed with concentrations of DMN ranging from 5 to 200 µM (at 100 µM NADPH) and NADPH as the electron donor, with concentrations ranging from 5 to 200 µM (at 150 µM DMN). Data were fitted by the Michaelis-Menten equation using a non-linear least-squares regression to determine *K*_*M*_ and *V*_max_ values for the reactions with NADPH and DMN. 2-n-Heptyl-4-hydroxyquinoline-N-oxide (Alexis Biochemicals) inhibition was tested in concentrations ranging from 1 to 100 µM. Data were fitted by the equation $v_{0}^{i}=v_{0}^{max}\frac{\left(1+\beta \frac{\left[I\right]}{K_{i}^{app}}\right)}{\left(1+\frac{\left[I\right]}{K_{i}^{app}}\right)}$, using a non-linear least-squares regression to determine *K*_*i*_^app^. The values presented are the median of at least three independent experiments. Non-linear least-squares regressions were performed with the tool solver from the Microsoft Office Excel program.

### Pre-steady-state kinetics experiments

The transient kinetics of NDH-2B reduction by NADPH and oxidation of reduced NDH-2B by DMN were studied by stopped flow using a TGK Scientific SF-61 DX2 apparatus placed inside an anaerobic chamber. The temperature of the drive syringes and mixing chamber was maintained at 15°C, and the pH was controlled with 50 mM MES Bis Tris Propane pH 5.5, 250 mM NaCl. All solutions were prepared inside the anaerobic chamber with degassed water. The time course of the reactions was monitored with a photodiode array (350–700 nm).

For the study of the reductive half reaction, 10 µM oxidized NDH-2B was mixed with 20 µM NADPH (1:2 ratio). To monitor the reduction of the protein over time, 100 spectra were acquired in 0.75 s. The rate constant was obtained from the fitting of the kinetic traces recorded at the wavelengths at which spectral changes were most evident (in this case, at 450 and 670 nm). Since a significant part of the reaction is not monitored due to the dead time of the apparatus (even at 15°C), the absorbance at time zero was taken from a control experiment in which NDH-2 was mixed with buffer. The time scale was corrected for the dead time (3 ms), and the fit of an exponential curve to the data was performed with the tool solver from the Microsoft Office Excel program.

For the study of the oxidative half reaction, 10 µM NDH-2B was reduced with 30 µM NADPH, and the reduced state of the enzyme was checked in a control experiment against buffer. Reduced NDH-2B was then mixed with 30 µM DMN in a 1:3 ratio. Another experiment was performed by mixing 10 µM of NDH-2B reduced with 50 µM NADPH with 30 µM of DMN (1:5:3 ratio) to allow complete turnover of the reaction. The software Kinetic Studio from TGK was used to fit exponential curves to the kinetic traces acquired at 450 and 670 nm. The values of the rate constants presented are the median of at least three independent experiments.

### Saturation transfer difference NMR

NMR spectra were acquired in a Bruker Avance III spectrometer at 15°C, operating at a proton frequency of 600.13 MHz with a 5 mm triple resonance cryogenic probe head. STD NMR spectra were acquired with 1,024 transients in a matrix with 32,000 data points in t2 in a spectral window of 12,019.23 Hz centered at 2,814.60 Hz. Excitation sculpting with gradients was employed to suppress the water proton signals. Selective saturation of protein resonances (on resonance spectrum) was performed by irradiating at −300 Hz using a series of 40 Eburp2.1000 shaped 90° pulses (50 ms, 1 ms delay between pulses), for a total saturation time of 2.0 s. For the reference spectrum (off resonance), the samples were irradiated at 20,000 Hz. Control experiments were performed with the reference samples in order to optimize the frequency for protein saturation (−0.5 ppm) and off-resonance irradiation to ensure that none of the tested ligands would be saturated at this frequency. STD spectra were obtained by subtraction of the off-resonance and the on-resonance spectra. The STD effect was calculated by (*I_0_ − I*_STD_)*/I_0_*, in which (*I_0_ − I*_STD_) is the peak intensity in the STD spectrum and *I_0_* is the peak intensity in the off-resonance spectrum. STD amplification factors were calculated by multiplying the STD effect by the ligand-protein ratio. For epitope mapping, the STD intensity of the largest STD effect was set to 100% as a reference, and the relative intensities were determined. The STD-NMR experiments were performed with NDH-2B in 50 mM MES pH 5.5, 250 mM NaCl buffer (prepared in D_2_O), and the different ligands of NADP^+^ (VWR) or DMN in the buffer (with 10% DMSO in the case of the quinone). The final concentrations of protein and ligands were 10 µM for NDH-2B, 0.5 mM for DMN, and 0.75 mM for NADP^+^.

### Fluorescence quenching studies

Fluorescence spectra were obtained on a Fluorolog Jobin Yvon, Horiba and a detector power source TBX-PS. The reaction mixture (400 µL) contained 2 µM NDH-2B in 25 mM MES Bis Tris Propane 250 mM NaCl pH 5.5. The effects of NADP^+^, NAD^+^ (Sigma-Aldrich), DMN, or HQNO on the protein were tested. Three independent titrations were performed with NADP^+^ and NAD^+^ (0–711 µM) and in the range of 0–250 µM with DMN and HQNO due to ligand solubility. For the titrations in the presence of HQNO, a concentration of 100 mM was used. Tryptophan fluorescence emission spectra were recorded at 25°C with excitation wavelengths at 280 nm for NADP^+^ or 295 nm for the DMN and HQNO. The change in emission at 330 nm (Δ*F*) was normalized and plotted vs ligand concentration. The values presented are the median of at least three independent experiments. Hyperbolic and Monod-Wyman-Changeux model equations were used to fit the data of NADP^+^ and NAD^+^ and DMN titrations, respectively. This was performed with the tool solver from the Microsoft Office Excel program.

### Bacterial strains and growth conditions

All strains and plasmids used in this study are listed in Table S6. Overnight cultures of NCTC8325-4 wild-type and knockout strains were diluted to OD_600nm_ = 0.05 in TSB (Table S7) and grown at 37°C with aeration. For growth and pH analyses, OD_600nm_ and pH measurements were taken every one and half hours for a total of 11 hours. For fluorescence microscopy experiments, overnight cultures of *S. aureus* strains were diluted to 1:200 in a fresh medium with appropriate inducers and allowed to grow until they reached an OD_600nm_ of approximately 0.6 before being collected and washed with phosphate-buffered saline (PBS). For metabolite NMR-based metabolomics analyses, samples (1.5 mL) were collected at 0, 3, and 6 hours of growth by centrifugation. The pellet was discarded, and the supernatant solutions were stored at −20°C. The handling of *S. aureus* was always performed inside a laminar flow chamber (NinoLaf Safety Cabinet, Modell ninoSAFE 1200).

### Construction of *S. aureus* mutants

The NDH-2A and NDH-2B knockout mutants were constructed using the pMAD vector (33) containing the upstream and downstream regions of each corresponding gene of interest to allow recombination and integration of the plasmids into the chromosome, followed by their excision with the genes to be deleted. Upstream and downstream regions of *ndh-2a* and *ndh-2b* genes were ampliﬁed by PCR, using primers P1_ndh-2A/P2_ndh-2A and P3_ndh-2A/P4_ndh-2A, P1_ndh-2B/P2_ndh-2B and P3_ndh-2B/P4_ndh-2B, respectively (Table S8). The PCR fragments encoding the upstream and downstream regions of each gene were joined by overlapping PCR using the pairs of primers P1_ndh-2A/P4_ndh-2A and P1_ndh-2B/P4_ndh-2B, respectively. The resulting fragments were digested with NcoI and BamHI (Fermentas) and cloned into previously digested pMAD vector and propagated in *E. coli* DC10B. The obtained inserts were sequenced. The plasmids were then electroporated into electrocompetent *S. aureus* RN4220 strain at 30°C, using erythromycin and X-gal selection, and transduced into NCTC8325-4 using phage 80*α* (34). The recombination and integration of the plasmids into the chromosome were performed as previously described (33) after a two-step homologous recombination process. In the first step, recombinants were selected at the non-permissive temperature of 43°C, using erythromycin and light blue colony color. In the second step, cells were incubated at the permissive temperature of 30°C in the absence of antibiotic selection, and white, erythromycin-sensitive colonies in which the vector had been excised were selected. Gene deletions were confirmed by PCR sequencing of the amplified fragment and the whole genome of the mutated strains. ∆*ndh-2a* and ∆*ndh-2b* strains lacking, respectively, NDH-2A and NDH-2B were obtained.

### *S. aureus* imaging by fluorescence microscopy

Super-resolution structured illumination microscopy (SIM) imaging was performed using an Elyra PS.1 microscope (Zeiss) with a Plan-Apochromat 63×/1.4 oil DIC M27 objective. SIM images were acquired using five grid rotations, with a 28 µm period for the 488 nm laser (100 mW). Images were captured using a Pco.edge 5.5 camera and reconstructed using ZEN software (black edition, 2012; version 8.1.0.484) based on a structured illumination algorithm, with synthetic, channel-specific optical transfer functions and noise filter settings ranging from −6 to −8.

To label *S. aureus* membranes, cells were incubated with Nile Red (Invitrogen) at a final concentration of 10 µg mL^−1^ for 5 min at room temperature, washed with PBS, and then mounted on microscope slides covered with a thin layer of agarose (1.2% in PBS). To calculate the volume of each cell, an ellipse was fitted to the border limits of the cellular membrane of Nile-Red-labeled cells, overlaying the membrane dye signal, using the ZEN microscopy software program. Subsequently, the shorter and longer axes were measured, coinciding with the septum and the axis perpendicular to it, respectively. The volume of the cell was obtained by an approximation to the volume of a prolate spheroid as described before (23).

### NMR-based metabolomics

Extracellular metabolome ^1^H-NMR analysis was performed in 5 mm glass tubes. For each collected sample, 400 µL was used and buffered to pH 7.0 by adding 200 µL of 200 mM sodium hydrogen phosphate buffer solution, prepared with 100% D_2_O. Additionally, the buffer solution contained a final concentration of 1 mM of TSP (3-trimethylsilyl-[2,2,3,3-D4]-1-propionic acid, Uvasol) as an internal standard for subsequent quantification, used for chemical shift referencing and normalization of NMR peak intensities. All ^1^H-NMR spectra were obtained at 25°C on a Bruker AVANCE III 500 NMR spectrometer, operating at a central frequency of 500.13 MHz (Bruker Biospin, Rheinstetten, Germany), equipped with 5 mm TCI C/N Prodigy Cryo probe. The equipment was controlled via Topspin version 3.2 software (Bruker Biospin, Rheinstetten, Germany), and spectra were acquired with pre-saturation for water signal suppression using a relaxation delay of 2 s. Twenty-seven spectra were collected. A total of 16 transients were collected into 64,000 data points using a spectral width of 16 ppm. Fourier transform was performed with no exponential multiplication. Spectra were automatically phased and baseline-corrected, and the chemical shift scale was set by assigning the value of *δ* = 0.00 ppm to the signal from the added TSP. Compound identification was done by matching the obtained spectra with a ^1^H-NMR spectra databank using Chenomx Nmr Suite Version 8.12 software (Chenomx Inc.) and comparing it with the spectra of standard compounds. Quantification was done by the integration of designated peaks, regarding the number of protons responsible for each signal in the defined frequencies (Table S9), and comparing with the added standard TSP (integral calibrated as nine protons, regarding the number of protons and the concentration in which they were present, 1 mM). The molar concentration of each compound was estimated considering the sample dilution (3:2). For each triplicate assay, the median was calculated, and the standard deviations were determined for all the metabolic peaks studied.

### Metabolomic flux distribution analysis

Exchange fluxes were calculated based on the rate of variation in extracellular concentration of metabolites (measured in the above-mentioned NMR experiments) estimated by the slopes of linear regressions. The slopes were obtained for every set of two consecutive time points (0/3 hours and 3/6 hours), using the media composition before culture inoculation as the 0-hour measurement and using 5 (0/3 hours) or 6 (3/6 hours) data points. The slopes for the 0/3-hour and 3/6-hour time intervals were normalized by the average OD of the respective strain growth curves during the corresponding time interval. The list of reactions present in the metabolic network of *Staphylococcus aureus* strain NCTC8325 was collected from the KEGG database using the KEGGREST R package. A metabolic graph for this strain was created where each metabolite was a vertex. Two metabolites were connected by an edge if they appeared in the same reaction equation, but one was a reactant while the other was a product. The metabolic graph was simplified by removing multiple edges between the same metabolites and removing metabolites that are (i) involved in a very large number (>133) of reactions (such as H_2_O and CO_2_), (ii) coenzymes (such as ATP and NADH), (iii) tRNAs, or (iv) do not correspond to a unique molecular species [such as thiocarboxy-(sulfur-carrier protein) and long-chain fatty acid]. A double network diffusion approach was applied to identify the metabolic reactions associated with the observed exchange fluxes and calculated respective reaction scores. An iterative diffusion method was used to estimate metabolite scores, according to the formula D(*n* + 1)= WAdj × *D*(*n*) + Input, where “*D*(*n*)” is a column vector of diffusion scores for all network nodes at iteration *n*, “WAdj” is a column degree normalized adjacency matrix of the metabolic graph, and “Input” is a column vector with the quantity of each node that is introduced into the network at each iteration. First, the absolute values of the exchange fluxes that were lower than 0 were used as input quantities for the corresponding metabolites in the iterative diffusion method. These source metabolites with negative exchange fluxes were being imported by cells. The iterative diffusion method will output a source diffusion score “Sdi” for every metabolite “i” in the network, quantifying how likely the diffusion from the source metabolites will reach each node (metabolite). Second, the exchange fluxes greater than 0 were also used as input quantities for the corresponding metabolites in the iterative diffusion method. These sink metabolites with positive exchange fluxes were being exported by cells. In this case, the method will output a sink diffusion score “Kdi” for every metabolite “i” in the network, quantifying how likely the diffusion from the sink metabolites will reach each node. To bias the network diffusion from the sources to the sinks (and *vice versa*), a second round of iterations was performed where the “WAdj” matrix was normalized by the diffusion scores of the first round (for the diffusion from the sources, “WAdj” was normalized by “Kdi” and for the diffusion from the sinks, it was normalized by “Sdi”). This means that in the biased diffusion (with sources or sinks as inputs), the flow out of a given node will be proportional to the connectivity of each neighbor to the preferred end nodes (sinks or source nodes, respectively). The number of iterations was defined as the maximal shortest path between input and output metabolites. In this way, the diffusion processes are allowed to reach all relevant metabolites that form pathways linking input and output metabolites.

For each edge in the network, connecting metabolites “i” and “j,” an edge score was computed as Esij = (Sdi × Kdj + Sdj × Kdi).

Finally, each reaction may be associated with multiple edges (reactions with more than one reactant or product). Therefore, a global score was computed for each reaction by selecting the related edge score with a maximum absolute value. To evaluate pathway enrichment, the top 50% of reactions with larger changes in reaction scores between the mutant and the wild-type strains were collected for each mutant. Random permutation tests were applied to identify KEGG pathways enriched in these sets of reactions. A total of 2,500 random samples of reaction sets (with the same size as the top 50% sets) were generated. For each random set, the number of reactions associated with each KEGG pathway was recorded. For each KEGG pathway, a *P*-value was computed. The *P*-value was the fraction of the random sets that showed an equal or higher number of associated reactions. Pathways with a *P*-value lower than 0.03 were considered as significantly enriched. Additionally, to avoid selecting pathways that were unrelated with the experimentally monitored external metabolites, we only considered pathways where at least one-third of its metabolites were part of the shortest paths linking these monitored external metabolites. The code used to generate the metabolic graph, reaction scores, minimal network, pathway enrichment, and related paper figures is available at https://github.com/GamaPintoLab/SAO_metabolic_network.

## SUPPLEMENTAL MATERIAL

Supplemental figures and tables spectrum.04152-23-s0001.pdf Fig. S1-S5; Tables S1-S9.

ASM does not own the copyrights to Supplemental Material that may be linked to, or accessed through, an article. The authors have granted ASM a non-exclusive, world-wide license to publish the Supplemental Material files. Please contact the corresponding author directly for reuse.
