# Supplementary material for: The two alternative NADH:quinone oxidoreductases from Staphylococcus aureus: two players with different molecular and cellular roles
Source: Microbiol Spectr. 2024 Jul 16;12(8):e04152-23. doi: 10.1128/spectrum.04152-23 (PMC11302666; doi:10.1128/spectrum.04152-23)
Supplement: Supplemental figures and tables — Fig. S1-S5; Tables S1-S9. [file spectrum.04152-23-s0001.pdf]

Supplementary Information

The two Alternative NADH:quinone oxidoreductases from *Staphylococcus aureus*: two players with different molecular and cellular roles

Filipa V. Sena[1,2], Filipe M. Sousa[1,2], Ana R. Pereira[2#], Teresa Catarino[2,3], Eurico J. Cabrita[3,4], Mariana G. Pinho[2], Francisco R. Pinto[1] and Manuela M. Pereira[1*]

[1]University of Lisbon, Faculty of Sciences, Department of Chemistry and Biochemistry and BioISI - Biosystems & Integrative Sciences Institute, Campo Grande, C8, 1749-016 Lisboa, Portugal

[2]Instituto de Tecnologia Química e Biológica – António Xavier, Universidade Nova de Lisboa, Av. da República EAN, 2780-157 Oeiras, Portugal

[3]Departamento de Química, Faculdade de Ciências e Tecnologia, Universidade Nova de Lisboa, 2829-516 Caparica, Portugal

[4]UCIBIO, Departamento de Química, Faculdade de Ciências e Tecnologia, Universidade Nova de Lisboa, 2829-516, Caparica, Portugal.

[5]Instituto de Medicina Molecular, Faculdade de Medicina da Universidade de Lisboa, 1649-028 Lisboa, Portugal (current address).

*Corresponding author E-mail: mmpereira@fc.ul.pt.

#Present address: Instituto de Medicina Molecular, Faculdade de Medicina da Universidade de Lisboa, 1649-028 Lisboa, Portugal.

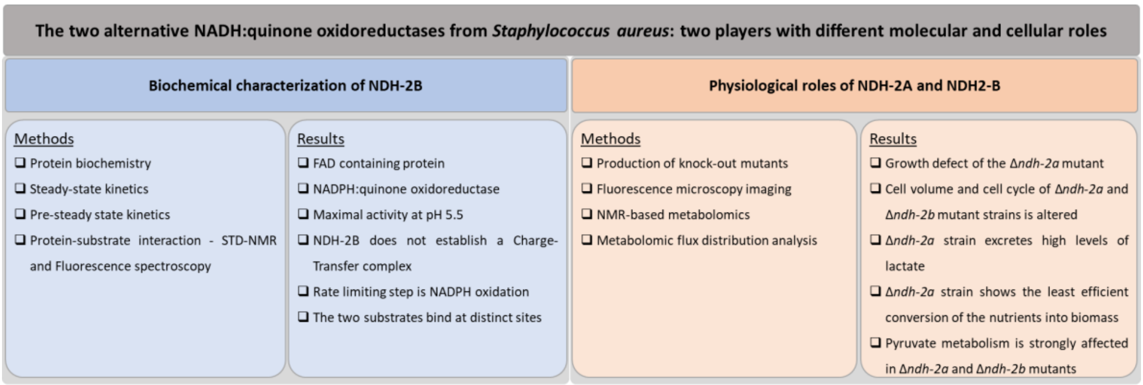

Figure S1. The two alternative NADH:quinone oxidoreductases from Staphylococcus aureus: two players with different molecular and cellular roles. Summary illustration of the work presented in this article, indicating the main methodologies performed and obtained results. An exhaustive biochemical characterization of NDH-2B was performed. We previously characterized NDH-2A (Sena et al 2015). The physiological role of NDH-2A and NDH-2B was investigated.

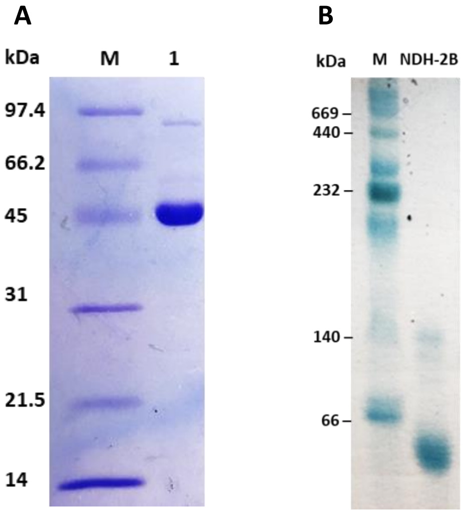

Figure S2. (A) SDS-PAGE gel of NDH-2B purified – lane 1. Theoretical molecular mass of NDH-2B: 40 KDa. Precision plus protein dual colour standard was used as marker. **(B) Blue native-PAGE gel of NDH-2B.** HMW calibration proteins from the calibration Kit for native electrophoresis was used to estimate the molecular mass of NDH-2B.

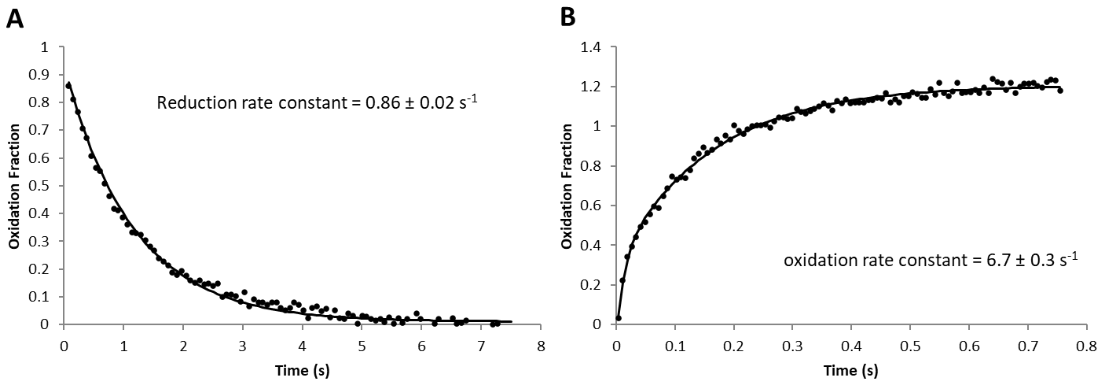

Figure S3. Pre-steady state analyses of NDH-2B from *S. aureus*. Oxidation fraction in the presence of **(A)** NADPH (1:2 ratio) and of **(B)** DMN (1:3 ratio). Oxidation factions were determined by the change in absorbance at 450 nm. The concentrations before mixing were 10 µM of NDH-2B, 20 µM of NADPH (A) and 30 µM for DMN (B) in a 50 mM MES Bis Tris Propane 250 mM NaCl buffer pH 5.5. The black dots represent the experimental data, and the line represents respective exponential fits.

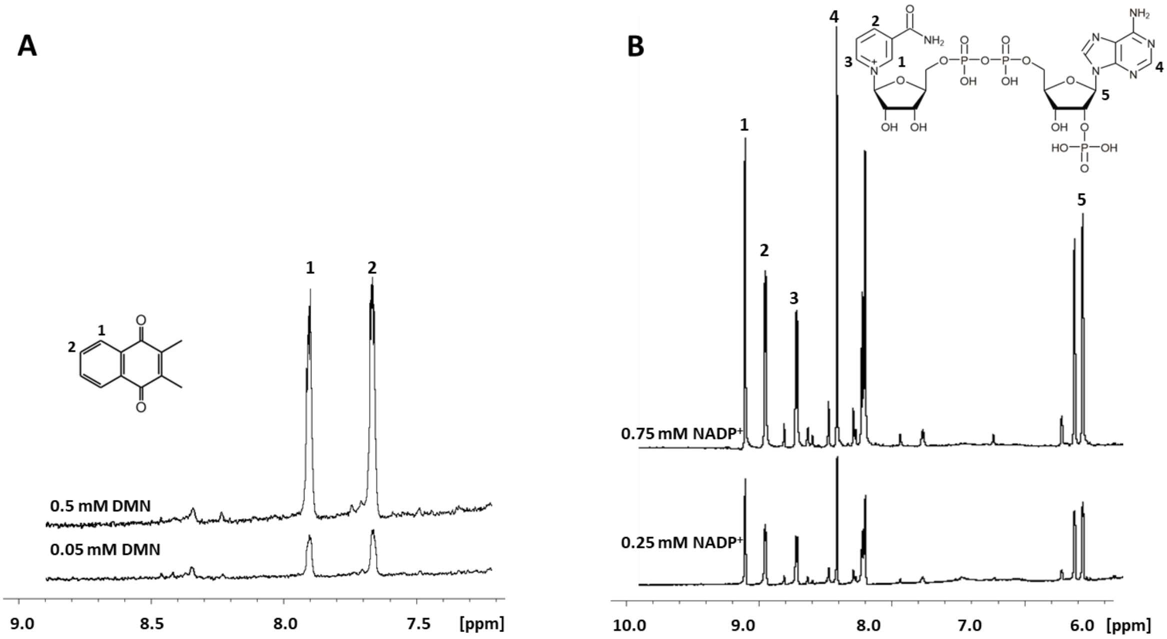

Figure S4. STD-NMR measurements with NDH-2B and substrates (DMN and NADP$^+$). (A) Illustrative STD-NMR spectra of NDH-2B titrated with DMN. **(B)** Illustrative STD-NMR spectra of NDH-2B titrated with NADP$^+$.

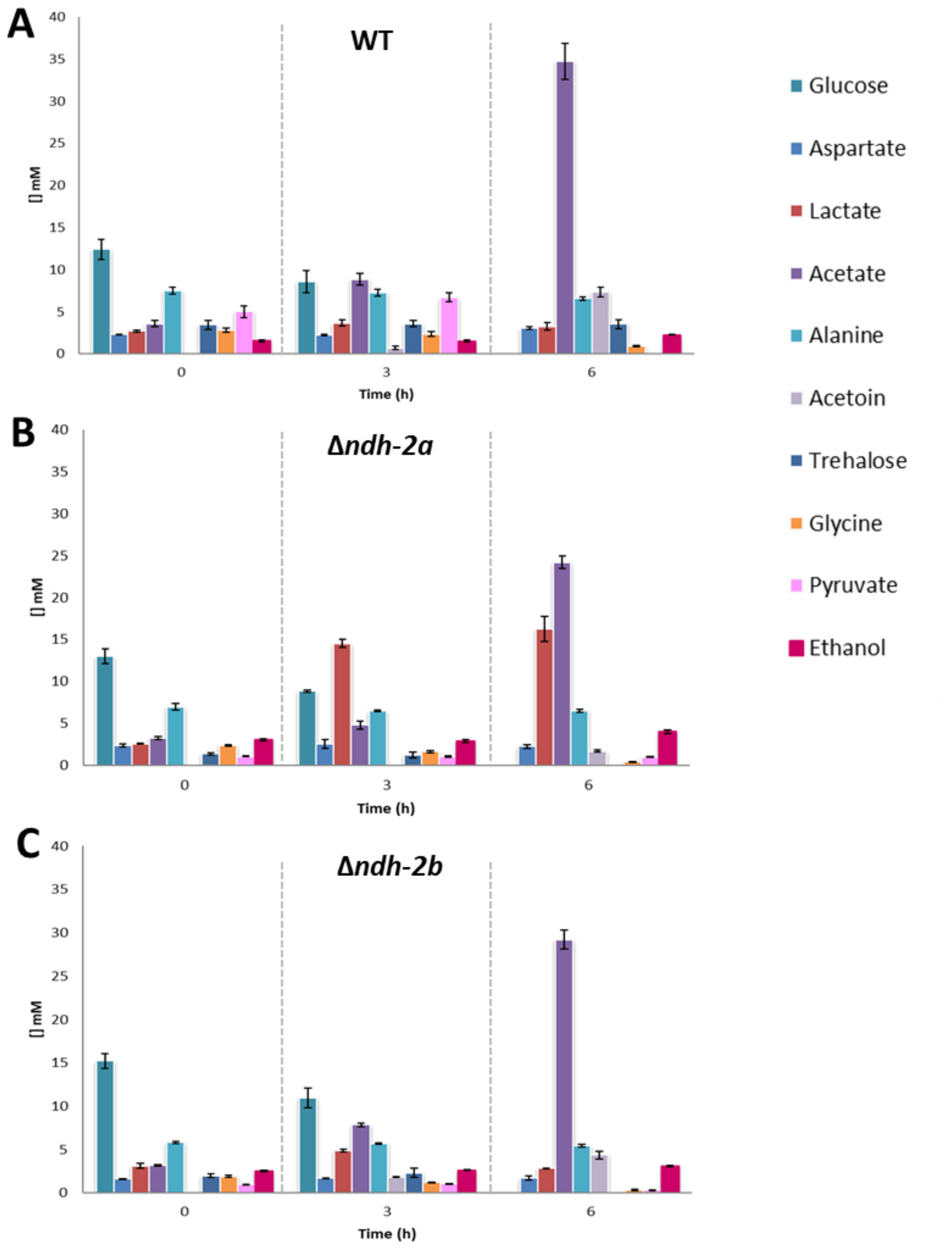

Figure S5. Extracellular metabolites identified by ^{1}H-NMR analysis. Bar Graphs representing the concentration of extracellular metabolites identified at the initial, 3 hours and 6 hours of growth *of S. aureus* strains: wild-type, Δ*ndh-2a* and Δ*ndh-2b*. Compound identification was done by matching the obtained spectra with a ^{1}H-NMR spectra databank and comparing with spectra of standard compounds. Quantification was obtained by integration of designated peaks (Table S9) and comparing with the added standard TSP (3-(trimethylsilyl)-propionic-acid sodium salt). Presented results were obtained from at least three independent measurements.

Table S1. Average ratio of longer/shorter axes of *S. aureus* cells upon the three phases of the cell cycle.

Strain	Volume (µm^3)		
	Phase 1	Phase 2	Phase 3
**WT**	0.47 ± 0.1	0.54 ± 0.1	0.64 ± 0.11
Δndh-2a	0.75 ± 0.14 ****	0.99 ± 0.14 ***	1.10 ± 0.15 ***
Δndh-2b	0.59 ± 0.13*	0.82 ± 0.13**	0.94 ± 0.13 ****

* p ≤ 0.05, ** p ≤ 0.01, *** p ≤ 0.001 and **** p ≤ 0.0001 mutants vs. parental strain NCTC (n=315 cells for each strain, n=105 cells per phase). Data are presented as mean ± standard deviation. Statistical analysis was performed using the unpaired *t test*.

Table S2. Exchange fluxes normalized by optical density.

Metabolite	0-3h			3-6h		
	wt	Δndh-2a	Δndh-2b	wt	*Δndh-2a*	*Δndh-2b*
	**Flux** (mM/h/OD)	**Flux** (mM/h/OD)	**Flux** (mM/h/OD)	**Flux** (mM/h/OD)	**Flux** (mM/h/OD)	**Flux** (mM/h/OD)
Trehalose (12C)	-0.337±0.169	-1.955±0.172***	-0.990±0.331	-0.012±0.039	-0.240±0.034**	-0.149±0.165
Glucose (6C)	-2.001±0.558	-2.478±0.079	-1.402±0.652	-0.939±0.077	-1.539±0.016***	-1.170±0.099
Pyruvate (3C)	0.627±0.294	-3.130±0.227***	-2.819±0.201***	-0.717±0.030	-0.016±0.011***	-0.066±0.018***
Lactate (3C)	0.738±0.184	7.796±0.241***	1.816±0.416	-0.058±0.036	0.402±0.160*	-0.314±0.065*
Acetate (2C)	1.041±0.361	-1.128±0.331**	1.134±0.708	2.615±0.135	3.402±0.092**	2.807±0.476
Ethanol (2C)	0.906±0.039	1.943±0.074***	1.387±0.205	0.069±0.006	0.172±0.025**	0.054±0.043
Acetoin (4C)	0.391±0.072	0.000±0.000*	1.182±0.157**	0.683±0.035	0.298±0.011***	0.434±0.167
Aspartate (4C)	-0.088±0.053	0.183±0.261	-0.286±0.082	0.085±0.012	-0.037±0.058	0.059±0.074
Methionine (5C)	-0.010±0.042	0.324±0.094*	-0.194±0.271	0.011±0.006	0.023±0.020	0.015±0.047
Alanine (3C)	-0.271±0.181	-0.856±0.031*	-1.008±0.290	-0.071±0.028	-0.002±0.018	0.033±0.112
Glycine (2C)	-0.629±0.127	-1.280±0.082**	-1.167±0.281	-0.165±0.018	-0.212±0.018	-0.089±0.068
Formate (1C)	-0.367±0.049	-0.479±0.051	-0.376±0.042	-0.003±0.010	0.051±0.013*	0.000±0.007
TMAO (3C)	-0.199±0.062	-0.624±0.057**	-0.515±0.125	-0.100±0.008	-0.095±0.007	-0.079±0.018
Dymethilamine(2C)	-0.199±0.007	-0.226±0.008*	0.138±0.106*	0.000±0.000	0.000±0.000	-0.002±0.028

*** p<0.001, ** p<0.01, * p<0.05, t test mutant vs parental strain.

Table S3. Exchange influxes, effluxes, and net fluxes (concentrations computed for a molecule with 3 carbon atoms)

Strain	**0-3h** (mM/h/OD)			**3-6h** (mM/h/OD)		
	**Metabolite influx**	**Metabolite efflux**	**Net flux**	**Metabolite influx**	**Metabolite efflux**	**Net flux**
**Wild type**	6.628±0.890	2.938±0.463	3.690±1.003	2.963±0.146	2.831±0.118	0.132±0.188
Δndh-2a	19.302±0.515***	7.277±0.404***	12.025±0.657***	4.342±0.102***	3.103±0.180***	1.239±0.207***
Δndh-2b	12.714±1.270**	4.560±0.759	8.154±1.479*	3.349±0.370	2.623±0.461**	0.727±0.592**

*** $p<0.001$, ** $p<0.01$, * $p<0.05$, t test mutant vs parental strain.

Table S4. NAD(P)H, NAD(P)$^+$ and net NAD(P)H productions

Strain	**0-3h** (mM/h/OD)			**3-6h** (mM/h/OD)		
	**NAD(P)H production**	**NAD(P)$^+$ production**	**Net NAD(P)H production**	**NAD(P)H production**	**NAD(P)$^+$ production**	**Net NAD(P)H production**
**Wild type**	5.350±0.858	2.097±0.190	3.253±0.879	1.985±0.134	0.103±0.007	1.882±0.139
Δndh-2a	12.776±0.362***	11.274±0.526***	1.502±0.638	4.039±0.072***	0.661±0.163***	3.378±0.178***
Δndh-2b	6.764±1.135	3.897±0.486*	2.867±1.234	3.248±0.364*	0.081±0.053**	3.167±0.368**

*** $p<0.001$, ** $p<0.01$, * $p<0.05$, t test mutant vs parental strain.

Table S5. Network diffusion algorithm. KEGG pathways with a higher number of reactions are listed in the top half of reactions with greater differences in the diffusion score relative to the wild type.

**Pathway**	**Number of reactions**	**% in top half with greater differences relative to wt** (p-value of test for difference from % expected by chance, that is 50%)	
		Δndh-2a	***Δndh-2b***
Pyruvate metabolism	28	100 (0.000)	100 (0.000)
Pentose phosphate pathway	24	92 (0.000)	29 (0.990)
Alanine, aspartate, and glutamate metabolism	20	90 (0,000)	100 (0.000)
Cysteine and methionine metabolism	28	79 (0.004)	89 (0.000)
Citrate cycle (TCA cycle)	17	76 (0.023)	94 (0.000)
Lysine biosynthesis	12	50 (0.618)	83 (0.019)

Table S6. Strains and plasmids used in this study.

Strains	Description	Source or reference
Escherichia coli		
DC10B	Δ*dcm* in the DH10B background; Dam methylation only; for cloning	(36)
Staphylococcus aureus		
RN4220	Restriction-deficient derivative of NCTC8325-4	(37)
NCTC8325-4	Wild-type strain (MSSA)	R. Novik
ΔNDH-2A	NCTC8325-4 with ndh-2A gene deleted	This work
ΔNDH-2B	NCTC8325-4 with ndh-2B gene deleted	This work
**Plasmids**	**Description**	**Source or reference**
pMAD	*E. coli-S. aureus* shuttle vector with a thermosensitive origin of replication for Gram positive bacteria; Ampr Eryr lacZ	(25)

Table S7. Tryptic Soy Broth (TSB) composition.

Compound	Amount (g/L)
Tryptone	17
K_2HPO_4	2.5
D-glucose	2.5
NaCl	5
Yeast extract	3

Table S8. Oligonucleotide primers used in this study. Bold sequences represent restriction cut sites.

Primer name	Nucleotide sequence (5'- 3')
p1pMAD-ndh2A_KO	TC**GGATCC**TCAACTGGAAAATGACCCAG
p2pMAD-ndh2A_KO	ACTTTATTTAAAGCTTAATTTCACCTAAGCTTTC
p3pMAD-ndh2A_KO	AATTAAGCTTTAAATAAAGTTTCAGCTAAAC
p4pMAD-ndh2A_KO	ATG**CCATGG**AACGACATTCGCAGCGCCACAC
p1pMAD-ndh2B_KO	TC**GGATCC**ACCTAAAAATTAAAATACAAATAG
p2pMAD-ndh2B_KO	GGTACTTCATTAATTAAAAAGCAATGTCAGTTC
p3pMAD-ndh2B_KO	CTTTTTAATTAATGAAGTACCCCTTTTATATG
p4pMAD-ndh2B_KO	ATG**CCATGG**TCAAATATGTCCTTAATCGTTATTG

Table S9. Compounds analyzed by ^{1H}-NMR with the chemical shifts and the number of protons.

Compound	Chemical Shift (ppm)		Protons
α-Glucose	5.233	Dup (3Hz)	1
β-Glucose	4.6441	Dup (7Hz)	
Aspartate	2.78	Dup	1
Methionine	2.12	Sing	3
Acetate	1.91	Sing	3
Alanine	1.47	Dup	3
Lactate	1.31	Dup(6.9Hz)	3
Acetoin	1.366	Dup	3
Trehalose	5.1864	Dup	1
Glycine	3.55	Sing	2
Pyruvate	2.36	Sing	3

Assignment performed based on the Chenomx Nmr Suite software and on the Biological Magnetic Resonance Data Bank.
